# Identification of FasL as a crucial host factor driving COVID-19 pathology and lethality

**DOI:** 10.1038/s41418-024-01278-6

**Published:** 2024-03-21

**Authors:** Marie-Christine Albert, Iratxe Uranga-Murillo, Maykel Arias, Diego De Miguel, Natacha Peña, Antonella Montinaro, Ana Beatriz Varanda, Sebastian J. Theobald, Itziar Areso, Julia Saggau, Manuel Koch, Gianmaria Liccardi, Nieves Peltzer, Jan Rybniker, Ramón Hurtado-Guerrero, Pedro Merino, Marta Monzón, Juan J. Badiola, Roman Reindl-Schwaighofer, Rebeca Sanz-Pamplona, Alberto Cebollada-Solanas, Zsolt Megyesfalvi, Balazs Dome, Maria Secrier, Boris Hartmann, Michael Bergmann, Julián Pardo, Henning Walczak

**Affiliations:** 1 Cell death, inflammation and immunity laboratory, CECAD Cluster of Excellence, University of Cologne, Cologne, 50931 Germany; 2 Cell death, inflammation and immunity laboratory, Institute of Biochemistry I, Centre for Biochemistry, Faculty of Medicine, University of Cologne, Cologne, 50931 Germany; 3 CIBER de Enfermedades Infecciosas, Instituto de Salud Carlos III, Madrid, 28029 Spain; 4 Aragón Health Research Institute (IIS Aragón), San Juan Bosco 13, Zaragoza, 50009 Spain; 5 Department of Microbiology, Paediatrics, Radiology and Preventive Medicine and Public Health, University of Zaragoza, Zaragoza, 50009 Spain; 6 Centre for Cell Death, Cancer, and Inflammation (CCCI), UCL Cancer Institute, University College London, London, WC1E 6DD UK; 7 Department I of Internal Medicine, Faculty of Medicine and University Hospital of Cologne, University of Cologne, Cologne, 50931 Germany; 8 Faculty of Medicine and University Hospital of Cologne, Centre for Molecular Medicine Cologne (CMMC), University of Cologne, Cologne, 50931 Germany; 9 German Centre for Infection Research (DZIF), Partner Site Bonn-Cologne, Cologne, 50931 Germany; 10 Genome instability, inflammation and cell death laboratory, Institute of Biochemistry I, Centre for Biochemistry, Faculty of Medicine, University of Cologne, Cologne, 50931 Germany; 11 Center for Molecular Medicine Cologne (CMMC), University of Cologne, Cologne, 50931 Germany; 12 Institue for Dental Research and Oral Musculoskeletal Biology, Faculty of Medicine and University Hospital Cologne, Cologne, 50931 Germany; 13 Department of Translational Genomics, University of Cologne, Cologne, 50931 Germany; 14 Instituto de Biocomputación y Física de Sistemas Complejos (BIFI), University of Zaragoza, Zaragoza, 50018 Spain; 15 Copenhagen Center for Glycomics, Department of Cellular and Molecular Medicine, University of Copenhagen, Copenhagen, 2200 Denmark; 16 Fundación ARAID, Zaragoza, 50018 Spain; 17 Research Centre for Encephalopaties and Transmissible Emerging Diseases, Institute for Health Research Aragón (IIS), University of Zaragoza, Zaragoza, 50013 Spain; 18 Department of Human Anatomy and Histology, University of Zaragoza, Zaragoza, 50009 Spain; 19 Department of Medicine III, Medical University of Vienna, Vienna, 1090 Austria; 20 CIBER de Epidemiología y Salud Pública, Instituto de Salud Carlos III, Madrid, 28029 Spain; 21 Aragon Biomedical Research Center (CIBA), Instituto Aragonés de Ciencias de la Salud (IACS), Unidad de Biocomputación, Zaragoza, 50018 Spain; 22 Deparment of Thoracic Surgery, Medical University of Vienna, Vienna, 1090 Austria; 23 Department of Thoracic Surgery, Semmelweis University and National Institute of Oncology, Budapest, 1122 Hungary; 24 National Koranyi Institute of Pulmonology, Budapest, 1121 Hungary; 25 Department of Translational Medicine, Lund University, Lund, SE-22100 Sweden; 26 UCL Genetics Institute, Department of Genetics, Evolution and Environment, University College London, London, WC1E 6BT United Kingdom; 27 Virology Group, Institute for Veterinary Disease Control at AGES, Moedling, 2340 Austria; 28 Div. of Visceral Surgery, Dept. of General Surgery, Comprehensive Cancer Centre, Medical University of Vienna, Vienna, 1090 Austria

**Keywords:** Cell death and immune response, Immunopathogenesis, Acute inflammation

## Abstract

The dysregulated immune response and inflammation resulting in severe COVID-19 are still incompletely understood. Having recently determined that aberrant death-ligand-induced cell death can cause lethal inflammation, we hypothesized that this process might also cause or contribute to inflammatory disease and lung failure following SARS-CoV-2 infection. To test this hypothesis, we developed a novel mouse-adapted SARS-CoV-2 model (MA20) that recapitulates key pathological features of COVID-19. Concomitantly with occurrence of cell death and inflammation, FasL expression was significantly increased on inflammatory monocytic macrophages and NK cells in the lungs of MA20-infected mice. Importantly, therapeutic FasL inhibition markedly increased survival of both, young and old MA20-infected mice coincident with substantially reduced cell death and inflammation in their lungs. Intriguingly, FasL was also increased in the bronchoalveolar lavage fluid of critically-ill COVID-19 patients. Together, these results identify FasL as a crucial host factor driving the immuno-pathology that underlies COVID-19 severity and lethality, and imply that patients with severe COVID-19 may significantly benefit from therapeutic inhibition of FasL.

## Introduction

Despite the unprecedentedly rapid development of effective vaccines, antiviral drugs and warning systems [1–3], severe acute respiratory syndrome (SARS) coronavirus (CoV) 2 (SARS-CoV-2), led to one of the most severe and deadly pandemics of the last centuries. Dismal disease outcome was due to severe lung failure, including acute respiratory distress syndrome (ARDS) [4, 5]. Murine models of mouse-adapted (MA) SARS-CoV and SARS-CoV-2 indicated that viral disease is characterised by a dysregulated type I, II and III interferon (IFN) response and subsequent lung organ failure based on inflammatory disease progression [6–8]. Correspondingly, SARS-CoV-2-induced lung failure is associated with an influx of neutrophils and inflammatory macrophages into the lungs, which is accompanied by an increase in cytokines and chemokines of the innate immune system [9, 10]. Unfortunately, current immunosuppressive treatments including dexamethasone, cytokine inhibition or Janus kinase blockade only provide limited therapeutic benefit to COVID-19 patients [11, 12]. This highlights the need for a better understanding of the pathophysiology of SARS-CoV-2-induced inflammation-associated lung failure.

In an independent line of research, we recently found that aberrant cell death induced by different death ligand members of the tumour necrosis factor (TNF) superfamily (TNFSF), including TNF, Fas ligand (FasL/CD95L) and TNF-related apoptosis-inducing ligand (TRAIL), can be causative for inflammatory processes resulting in lethal organ dysfunction [13–17]. Interestingly, in addition to an inappropriate innate immune response, untoward alveolar epithelial cell death had previously been proposed to be involved in the pathogenesis of ARDS [18, 19], and this concept was recently extended to COVID-19-associated lung damage [20–22]. Regarding death ligands, FasL was shown to be upregulated in lung tissue of ARDS patients [23] and treatment with exogenous FasL killed alveolar epithelial cells and induced ARDS in rabbits [24]. More recently, a positive correlation between sFasL levels in the plasma of COVID-19 patients with lymphopenia, CD4^+^ T susceptibility to apoptosis and disease severity was reported, suggesting that FasL might be involved in the lymphopenia characteristic of COVID-19 and could be a marker for COVID-19 severity [25–27]. In addition, non-canonical Fas-FasL signalling was proposed to be involved in SARS-CoV-2-induced macrophage activation and dysfunction [28]. It remains unresolved, however, whether or not FasL plays a causal role in COVID-19-associated lung damage and deaths [4, 5].

Using a newly developed mouse model of COVID-19, capable of inducing severe lung pathology in mice and resembling COVID-19 in humans, we identify the induction of cell death and inflammation and the expression of FasL to be highly associated with disease. Therapeutic inhibition of FasL, mainly expressed by lung NK cells and inflammatory monocyte/macrophages, markedly reduced cell death and inflammation in the lungs of both, young BALB/c or aged C57BL/6 mice and significantly increased their survival, importantly, without altering viral titres. Taken together, we identify a prominent role for FasL as a previously unrecognised key contributing factor to the inflammatory immunopathology characteristic of severe COVID-19 and propose, with the inhibition of FasL, a potential novel therapeutic opportunity for this disease.

## Results

### Mouse-adapted SARS-CoV-2 induces severe disease in young BALB/c and aged-to-old C57BL/6 mice

**Fig. 1:**
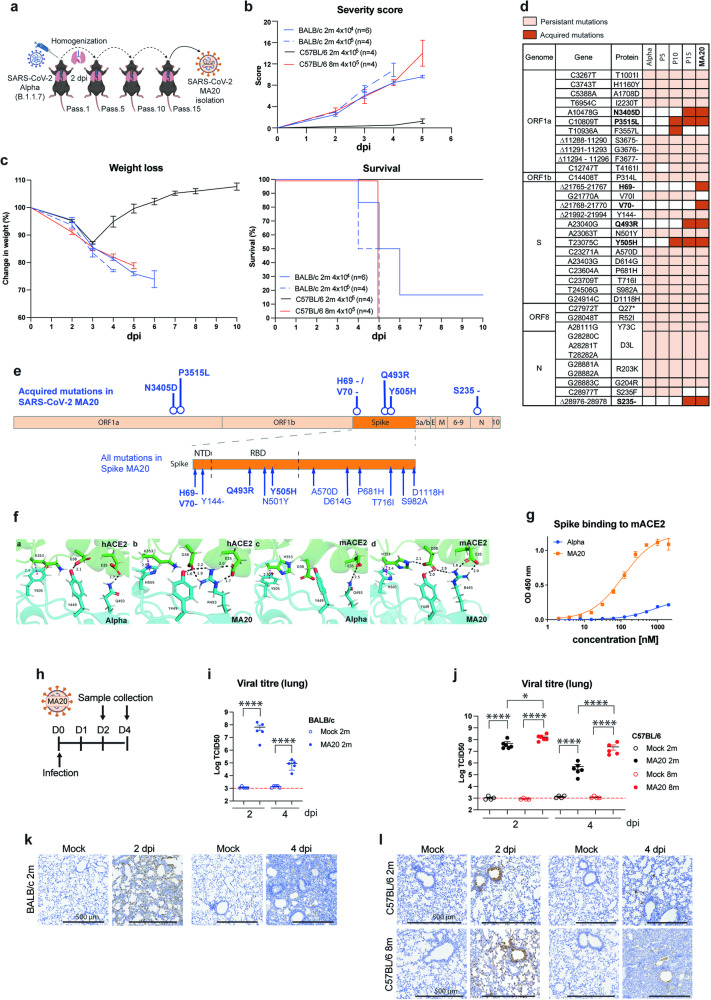
Mouse-adapted SARS-CoV-2 induces lethal disease. **a** Illustration of mouse adaptation process with SARS-CoV-2 Alpha variant by serial passages (pass.) to obtain SARS-CoV-2 MA20. Severity score (**b**), weight loss curves (**c**, left) and survival curves (**c**, right) of 2 m BALB/c, 2 m C57BL/6 and 8 m C57BL/6 mice infected with indicated viral titres of MA20(*n* = 4 or 6). **d** Persistent (light orange) and acquired (dark orange) mutations in SARS-CoV-2 isolates during mouse adaptation as compared to SARS-CoV-2 Wuhan strain. **e** Location of mutations within genes of SARS-CoV-2 MA20, acquired mutations depicted in bold. **f** (a–d) Depiction of predicted interactions with H-bonds (black) and CH-π interactions (magenta) of Alpha Spike RBD (cyan) in complex with hACE2 (green) (a), MA20 Spike RBD (cyan) in complex with hACE2 (green) (b), Alpha Spike RBD (cyan) in complex with mACE2 (green) (c), MA20 Spike RBD (cyan) in complex with mACE2 (green) (d). Only interactions up to 3.5 Å are shown. For H-bonds indicated distances correspond to distance between the corresponding hydrogen atom and acceptor oxygen atoms of either D38 or E35. **g** ELISA-style binding assay of the RBD of Spike proteins from Alpha (blue) and MA20 (orange) with mACE2 (*n* = 3). **h** Experimental design for sample collection after infection with MA20. Viral titres of infected 2 m BALB/c (**i**), 2 m C57BL/6 and 8 m C57BL/6 (**j**) mice (*n* = 4 to 6). *p* values were determined by One-way ANOVA with post-hoc Tukey.*p < 0.0332, **p < 0.0021, ***p < 0.0002, ****p < 0.0001. IHC staining of lung sections of SARS-CoV-2 Spike protein of infected 2 m BALB/c (**k**), 2 m C57BL/6 and 8 m C57BL/6 (**l**) mice. Values represent mea*n* ± SEM. Scale bars indicate 500 µm. 2 m:2-month-old. 8 m: 8-month-old.

To generate a pathogenic mouse-adapted strain of SARS-CoV-2 we used a clinical isolate of the SARS-CoV-2 Alpha variant (Pango Nomenclature B.1.1.7) [29] as a parental strain for mouse adaptation. This strain contained the N501Y mutation within the Spike protein which had, at the time, been suggested to enhance the affinity of the Spike protein to murine Angiotensin-converting enzyme (ACE2) (mACE2) [30, 31]. To obtain a virus strain that would not only infect mice but also induce severe disease upon infection, we performed 20 serial passages in one-year-old C57BL/6 mice since, as in humans, older mice are also more susceptible to viral infection (Fig. 1a). This procedure resulted in the generation of a mouse-adapted SARS-CoV-2 strain which we termed MA20. Viral titration of the intermediate passages showed steady titres up to passage 15 when viral titres increased (Supplementary information, Fig. S1a). The analysis of MA20-infected mice revealed that this newly isolated strain was capable of inducing severe disease and lethality in mice (Fig. 1 and Supplementary information, Fig. S1). In contrast, mice infected with early passages, as the exemplified Passage 7 virus (Supplementary information, Fig. S1b), did not lose weight and did not show any sign of disease.

We first performed viral titrations and determined disease severity and survival of young (2-month-old) BALB/c and C57BL/6 mice as well as aged (5-month-old, 8-month-old) and old (11-month-old) C57BL/6 mice upon MA20 infection (Fig. 1b, c and Supplementary information, Fig. S1c–i). In all cases, increased disease severity and decreased survival were proportional to the viral dose used for infection and dependent on mouse strain and age as shown for C57BL/6 mice (Fig. 1b, c and Supplementary information, Fig. S1c, i). Whereas infected young BALB/c mice showed severe signs of disease (significant weight loss and 100% lethality) at a viral dose of 4 × 10^4^ TCID50, young C57BL/6 mice only developed mild symptoms (with approx. 10% weight loss and complete survival) even at a 10-fold higher viral dose (Fig. 1b, c and Supplementary information, Fig. S1c–e). Yet, similar to young BALB/c mice, aged, and old C57BL/6 mice experienced an age-dependent decrease in survival and increased severe disease when exposed to infection by 4 × 10^4^ TCID50 and 4 × 10^5^ TCID50 of MA20 (Fig. 1b, c and Supplementary information, Fig. S1c and S1f-i). These results agree with other viral infection models where it was found that young BALB/c mice and aged C57BL/6 mice are more susceptible to infection and develop more severe disease than young C57BL/6 mice [32, 33]. Importantly, regardless of the mouse strain, in all severe cases mice showed clear respiratory symptoms, along with reduced activity, ruffled fur and hunched back (Fig. 1b). The selected viral clone used for infection was analysed by deep sequencing along with samples corresponding to intermediate passages (P5, P10, P15) to track the mutational landscape during the adaptation process. This revealed that with ΔH69V70, Q493R and Y505H, 4 of the 7 acquired mutations of the MA20 isolate compared to the original Alpha variant isolate of SARS-CoV-2 were located within the Spike protein (Fig. 1d, e).

To characterise the mechanism involved in the increased pathogenicity of MA20, and particularly the role of MA20 receptor binding domain (RBD) mutations, we performed molecular dynamics simulations using the structure of the SARS-CoV-2 RBD protein bound to human ACE2 (hACE2) receptor as a template [34]. Molecular Dynamics (MD) simulations with hACE2 and mACE2 complexed to either Alpha or MA20 Spike RBDs showed stability throughout the simulation period (Supplementary information, Fig. S2a). A closer look revealed that Y505H had little contribution to the binding to ACE2 when compared to the Q493R mutation (Fig. 1f). The Q493R mutation in MA20 established electrostatic interactions with Asp38 (D38) and Glu35 (E35) in ACE2, and favoured a hydrogen bond between Tyr499 (Y499) and Asp38 in the mACE2-MA20 RBD complex when compared to the other combinations (Supplementary information, Fig. S2b, c). Binding assays using recombinant proteins consisting of the N-terminal domain (NTD) and the RBD of the Spike proteins of SARS-CoV-2 Alpha or MA20, with mACE2, confirmed the performed simulations as the binding affinity to mACE2 was significantly enhanced for MA20 (Fig. 1g and Supplementary information, Fig. S2d). Of note, the RBD of SARS-CoV-2 Alpha also bound to mACE2, albeit only weakly, indicative of the capability of this variant to initiate the infectious process required for mouse adaptation. Collectively, these analyses explain the enhanced affinity of the Spike protein of MA20 to mACE2 as compared to that of the Alpha variant.

In order to quantify the presence of the virus during the course of infection in both BALB/c and C57BL/6 mice, we infected young (2-month-old) BALB/c mice as well as young and aged (8-month-old) C57BL/6 mice with MA20 and determined the presence of the virus in the lungs at 2 and at 4 days post infection (dpi) (Fig. 1h). Titres of infectious virus were highest at 2 dpi in all mouse strains tested and declined at 4 dpi (Fig. 1i, j). Additionally, aged C57BL/6 mice, which developed a more severe disease, also revealed higher viral titres already at 2 dpi as compared to young C57BL/6 mice with the difference between these two groups being further pronounced at 4 dpi (Fig. 1j). As demonstrated by immunohistochemistry (IHC) staining of the Spike protein, MA20 could readily be detected in bronchial tissue at 2 dpi and with mainly an alveolar distribution at 4 dpi (Fig. 1k, l). Of note, viral presence was not observed in the brains of infected mice by IHC (Supplementary information, Fig. S2e), in accordance with other previously described mouse-adapted SARS-CoV-2 models [7, 33, 35]. Thus, with the isolation of MA20 we established a new mouse model of SARS-CoV-2 infection which showed an age-dependent severity, resembling the situation in humans [36].

### Pathology induced by SARS-CoV-2 MA20 in mice recapitulates decisive features of human COVID-19

**Fig. 2:**
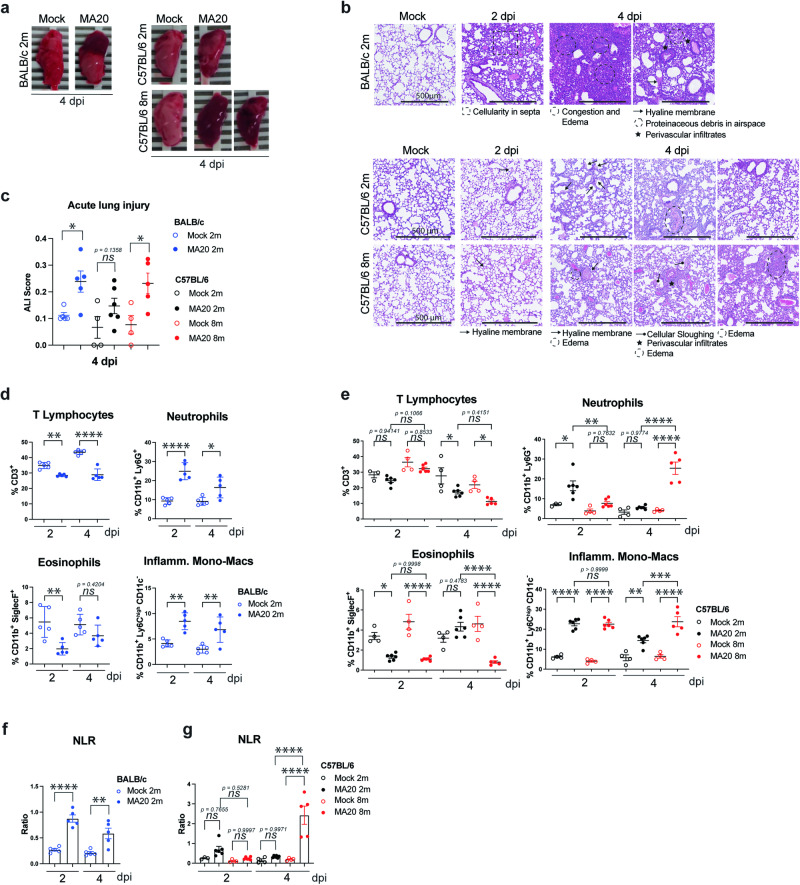
MA20-induced pathology resembles COVID-19. **a** Macroscopic pictures of left lung lobes at 4 dpi of infected 2 m BALB/c, 2 m C57BL/6 and 8 m C57BL/6 (right). **b** Histological Pathology of H&E-stained lung sections of infected 2 m BALB/c (upper panel), 2 m C57BL/6 (middle panel) and 8 m C57BL/6 (lower panel) mice at 2 dpi and 4 dpi with indicated prominent pathological features. **c** ALI score at 4 dpi of infected mice as indicated (*n* = 4–6). *p* values were determined by unpaired t-test with Welch’s correction. *p < 0.0332, **p < 0.0021, ***p < 0.0002, ****p < 0.0001. Flow cytometry analysis of immune cell populations in lungs of infected 2 m BALB/c, (**d**) 2 m C57BL/6 and 8 m C57BL/6 (**e**) mice as indicated (*n* = 3 to 6). *p* values were determined by One-way ANOVA with post-hoc Tukey. *p < 0.0332, **p < 0.0021, ***p < 0.0002, ****p < 0.0001. NLR of MA20-infected 2 m BALB/c (**f**), 2 m C57BL/6 and 8 m C57BL/6 (**g**) mice. *p* values were determined by One-way ANOVA with post-hoc Tukey (*n* = 3–6). *p < 0.0332, **p < 0.0021, ***p < 0.0002, ****p < 0.0001. Values represent mea*n* ± SEM. Scale bars indicate 500 µm. ALI acute lung injury. Inflamm. Mono-Macs inflammatory monocytic-macrophages. NLR neutrophil/lymphocyte ratio.2 m 2-month-old. 8 m 8-month-old.

We next examined the lungs of young BALB/c and young and aged C57BL/6 mice at different times following infection by MA20 (2 and 4 dpi). Especially at 4 dpi, gross damage to the lungs of severely-ill mice, with large areas of internal bleeding, was macroscopically evident (Fig. 2a). Lung weight of severely ill, MA20-infected young BALB/c and aged C57BL/6 mice increased significantly after infection, suggesting vascular leakage, oedema and immune cell infiltration, whereas this was not observed in young C57BL/6 mice which only developed mild disease (Supplementary information, Fig. S3a, b). Histopathological analysis revealed the establishment of hyaline membranes, perivascular infiltrates as well as oedema being most prominent at 4 dpi in young BALB/c and aged C57BL/6 mice, whereas young C57BL/6 mice only showed marginal signs of lung tissue alterations as quantified by determining the acute lung injury (ALI) score (Fig. 2b, c).

We next analysed the abundance of different immune cell populations in lung homogenates by flow cytometry. In line with observations in COVID-19 patients [37–39], we observed alterations in several immune cell populations, including a decrease in T lymphocytes (lymphopenia) and eosinophils (eosinopenia) as well as an increase in neutrophils (neutrophilia) already 2 dpi in both, BALB/c and C57BL/6 mice (Fig. 2d, e and Supplementary information, Fig. S3c–f). Although young and aged C57BL/6 mice showed a similar trend in immune cell dysregulation early after infection, this dysregulation resolved significantly faster in young C57BL/6 mice, as in these mice neutrophil and eosinophil numbers returned to mock infection levels at 4 dpi, whereas in aged mice their numbers remained pathologically altered at this time (Fig. 2e). Accordingly, the neutrophil to lymphocyte (NLR) and eosinophil to lymphocyte (ELR) ratios also revealed significant changes (increase in NLR and decrease in ELR) in BALB/c as well as aged C57BL/6 mice, but not in young C57BL/6 mice (Fig. 2f, g and Supplementary information, Fig. S3g,h). Inflammatory Monocytic-Macrophages (IMMs) and their increased presence in the lungs of infected mice were previously shown to promote lethality in a mouse-adapted model of SARS-CoV infection [7]. When analysing the effect of MA20 infection on IMMs, we found that their presence was significantly increased at 4 dpi in the lungs of young BALB/c and aged C57BL/6 mice as compared to young C57BL/6 mice (Fig. 2d, e). Thus, the prolonged dysregulation of cell infiltrates at 4 dpi correlates with lung injury and reflects disease outcome. Furthermore, we observed a strong increase in NK cells, whereas CD4+ and CD8+ T cells were diminished in BALB/c mice (Supplementary information, Fig. S3e) and a similar trend was observed in C57BL/6 mice (Supplementary information, Fig. S3f). Again, the reduction of CD4+ and CD8+ T cells was more pronounced in aged than in young C57BL/6 mice. Taken together, the severe pathophysiology caused by MA20 infection in young BALB/c and aged C57BL/6 mice recapitulates key features of the pathophysiology of severe COVID-19 in humans.

### SARS-CoV-2 MA20 promotes lung cell death and upregulation of cell death-related genes

**Fig. 3:**
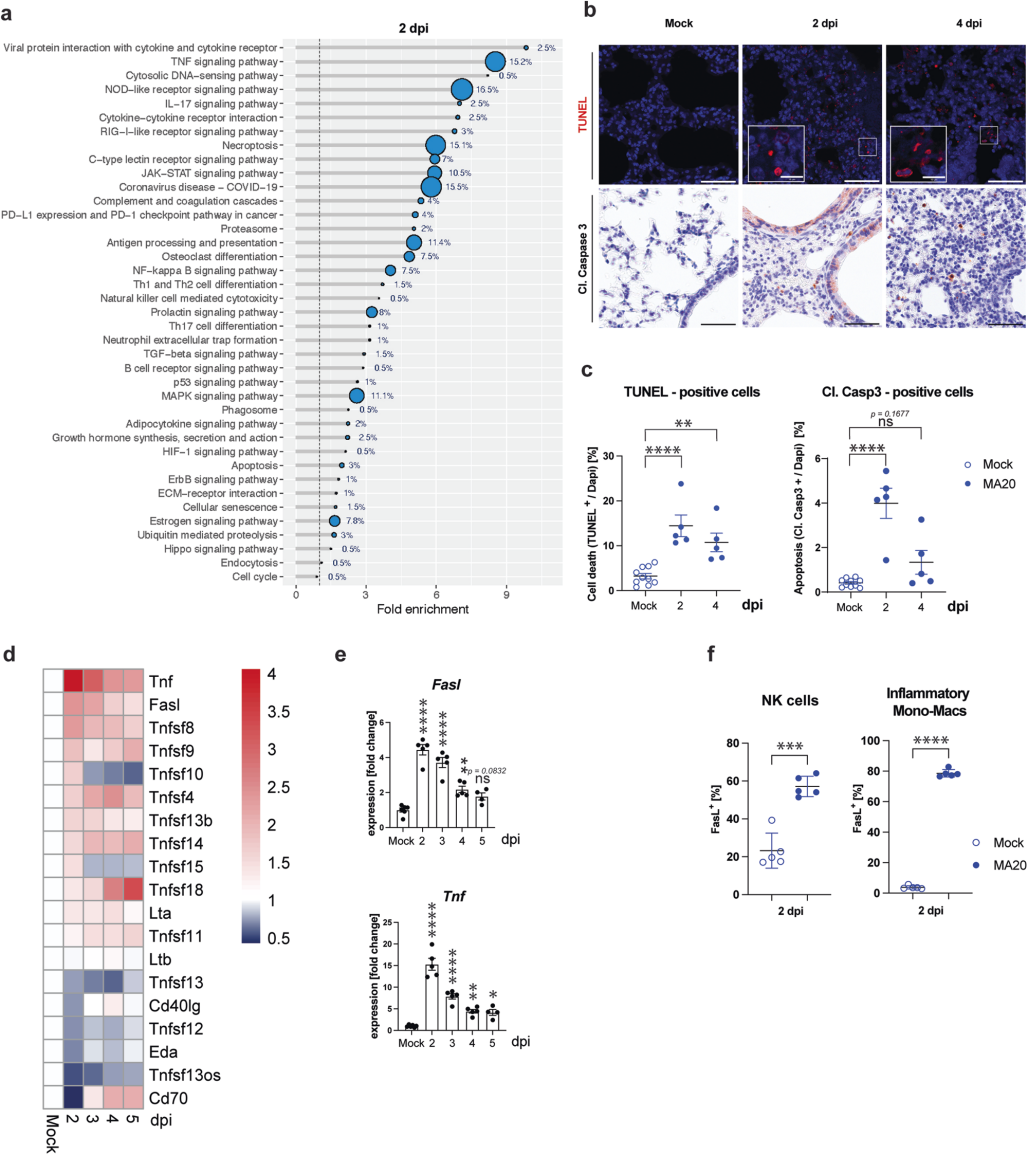
MA20 induces cell death in infected lungs. **a** Pathway ontology analysis of RNAseq results of lungs of infected 2 m BALB/c mice at 2 dpi. Percentage indicates number of genes dysregulated in respective pathway. Representative pictures (**b**) and quantification (**c**) of TUNEL-stained and cleaved Caspase 3 staining of lung sections of infected 2 m BALB/c mice at 2 and 4 dpi (*n* = 5 or 10). *p* values were determined by One-way ANOVA with post-hoc Dunnett. *p < 0.0332, **p < 0.0021, ***p < 0.0002, ****p < 0.0001. **d** RNAseq expression analysis of TNFSF members of lungs of infected 2 m BALB/c mice (*n* = 4 to 5). Data are represented as log2 fold change relative to mock. **e** Individual expression values of *Fasl* (left panel) and *Tnf* (right panel) (*n* = 5 to 6). *p* values were determined by One-way ANOVA with post-hoc Dunnett. *p < 0.0332, **p < 0.0021, ***p < 0.0002, ****p < 0.0001. **f** Flow cytometry analysis of FasL expression on immune cell populations as indicated in infected 2 m BALB/c mice at 2 dpi (*n* = 5). *p* values were determined by unpaired t-test with Welch’s correction. *p < 0.0332, **p < 0.0021, ***p < 0.0002, ****p < 0.0001. Scale bars indicate 50 µm in overview and 10 µm in enlarged frames. Values represent mea*n* ± SEM. NK cells: Natural Killer cells. Inflammatory Mono-Macs inflammatory monocytic-macrophages, 2 m 2-month-old.

To assess which signalling pathways were dysregulated on course of MA20-infection-caused severe disease, we performed transcriptomic analysis by bulk RNA sequencing at 2, 3, 4 and 5 dpi of the lungs of young BALB/c mice infected with 4 × 10^4^ TCID50 of MA20. Cluster analysis revealed that the overall RNA signature differed strikingly between mock- and MA20-infected mice (Supplementary information, Fig. S4a). Intriguingly, cell death-related pathways such as ‘TNF signalling’, ‘necroptosis’ and ‘apoptosis’ were amongst the most highly dysregulated pathways upon MA20 infection (Fig. 3a).

To determine whether untoward cell death may play a role in disease manifestation following MA20 infection, we next analysed the extent of cell death in lung samples of infected BALB/c mice. In comparison to mock-infected mice, we observed significantly more Terminal deoxynucleotidyl transferase dUTP nick end labelling (TUNEL)-positive cells at 2 dpi (Fig. 3b, c and Supplementary information, Fig. S4b). Additionally, we studied the contribution of caspase activation to the cell death detected in the lungs and found a significant increase in cleaved Caspase 3-positive cells upon infection (Fig. 3b, c). Thus, considering our previous findings showing that cell death induced by different death ligands can have up to lethal pathological consequences by triggering severe inflammation [13–15, 17], we next determined which members of the TNFSF were upregulated. This revealed that, amongst all members of this family, there was a highly significant increase in the expression of *Tnf* and *Fasl* in MA20-infected mice at 2 and 3 dpi as compared to mock-infected controls (Fig. 3d, e). Whilst TNF was previously shown to be implicated in severe COVID-19, a potential role of FasL in the development of severe disease following infection by SARS-CoV-2 has not been assessed [40, 41].

To investigate which immune cells mainly contributed to FasL production, we analysed its expression on the surface of different lung immune cell populations by flow cytometry. This revealed that FasL expression was highly upregulated on the surface of IMMs and NK cells (Fig. 3f and Supplementary information, Fig. S4c), whose presence was also significantly increased in the lungs of MA20-infected mice upon disease progression (Fig. 3d, e). Together, these results support the hypothesis that FasL could be responsible for, or at least contribute to, aberrant lung cell death triggered in response to infection by MA20 and, thereby, contribute to inflammatory disease severity and lethality.

### Therapeutic inhibition of FasL decreases disease severity and lethality following MA20 infection

**Fig. 4:**
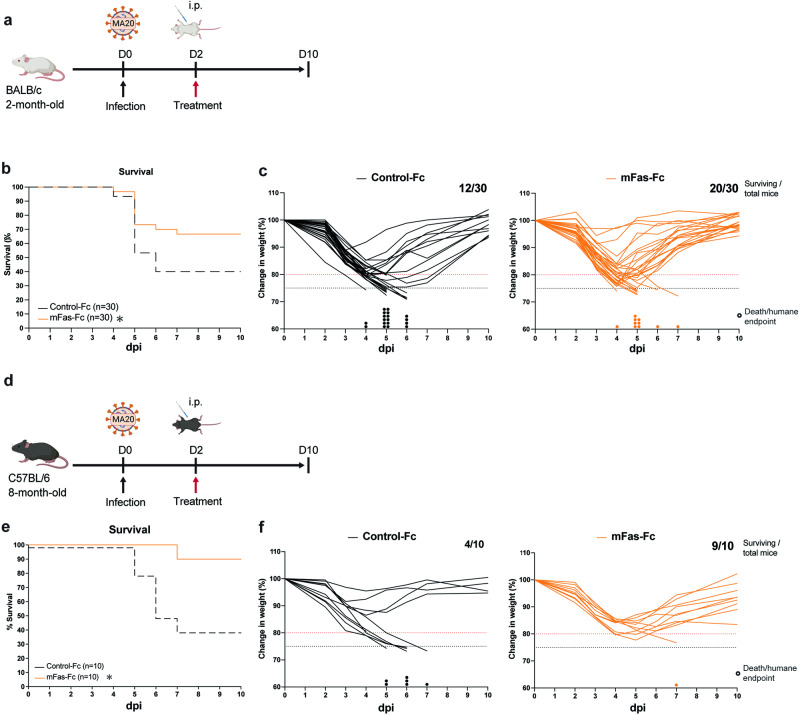
Therapeutic FasL inhibition prevents lethality in MA20-infected mice. **a** Experimental design for i.p. treatment after infection of 2 mBALB/c mice. Survival curves (**b**) and single weight loss curves (**c**) of MA20-infected 2m BALB/c mice with i.p. injections at 2 dpi with indicated treatments (*n* = 30). Coloured dots indicate when mice reached humane endpoint. Survival of mice with either treatment was compared by log-rank test. *p < 0.0332, **p < 0.0021, ***p < 0.0002, ****p < 0.0001. **d** Experimental design for i.p. treatment after infection of 8 m C57BL/6 mice. Survival curves (**e**) and single weight loss curves (**f**) of MA20-infected 8 m C57BL/6 mice with i.p. injections at 2 dpi with each indicated treatments (*n* = 10). Coloured dots indicate when mice reached humane endpoint. Survival of mice with either treatment was compared by log-rank test. *p < 0.0332, **p < 0.0021, ***p < 0.0002, ****p < 0.0001. D Day. i.p. intraperitoneal, 2 m 2-month-old. 8 m 8-month-old.

We next determined whether the therapeutic inhibition of FasL may alleviate the severe pathology induced by MA20 infection. We therefore treated MA20-infected young BALB/c mice at 2 dpi with mouse Fas-Fc (mFas-Fc) and measured overall survival and weight loss over time (Fig. 4a). We chose 2 dpi for the treatment since MA20-infected mice showed first overt signs of falling ill at this time and because this time is relevant for use in symptomatic COVID-19 patients. Strikingly, the inhibition of FasL significantly increased survival of MA20-infected mice when compared to Control-Fc-treated mice, with overall survival rising from 40 to 67% (Fig. 4b, c). Importantly, treatment of aged C57BL/6 mice 2 days following infection by MA20 with mFas-Fc provided a significant survival advantage also in these mice, with overall survival improving from 40 to 90% (Fig. 4d–f). These results demonstrate that therapeutic inhibition of FasL provides benefit to MA20-infected mice by significantly reducing the severity of the disease they develop and, consequently, significantly increasing their survival.

### Therapeutic inhibition of FasL diminishes cell death and inflammation in the lungs of MA20-infected mice

**Fig. 5:**
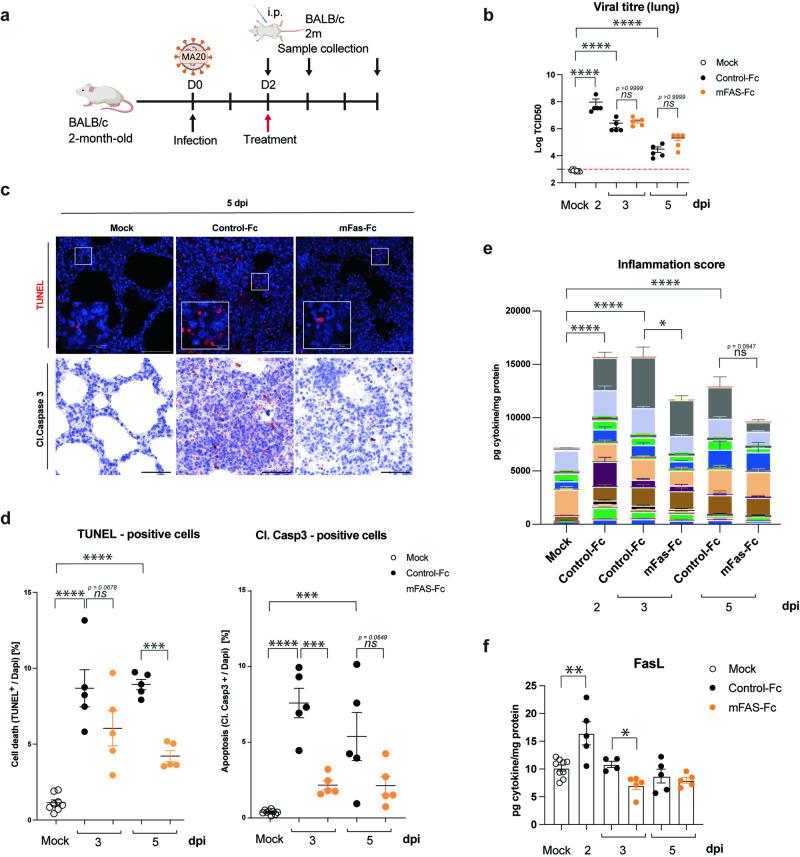
Inhibition of FasL decreases overall cell death and inflammation in infected lungs. **a** Experimental design for i.p. treatment and sample collection after infection. **b** Viral titres of lungs of infected 2 m BALB/c mice with indicated treatments (*n* = 9 or 5). *p* values were determined by One-way ANOVA with post-hoc Tukey. *p < 0.0332, **p < 0.0021, ***p < 0.0002, ****p < 0.0001. Representative pictures (**c**) and quantification (**d**) of TUNEL and cleaved caspase 3 staining of lung sections of infected 2 m BALB/c mice with indicated treatments (*n* = 9 or 5). *p* values were determined by One-way ANOVA with post-hoc Dunnett. *p < 0.0332, **p < 0.0021, ***p < 0.0002, ****p < 0.0001. **e** Inflammation score depicting accumulation of 39 cytokines and chemokines quantified by Luminex Multiplex Assay normalized to protein amount in lung homogenates of infected 2 m BALB/c mice with indicated treatments (*n* = 9 or 5). *p* values were determined by Two-way ANOVA with post-hoc Tukey. *p < 0.0332, **p < 0.0021, ***p < 0.0002, ****p < 0.0001. **f** Protein levels of FasL in lung samples of infected 2 m BALB/c mice with indicated treatments (*n* = 9 or 5). *p* values were determined by One-way ANOVA with post-hoc Dunnett. *p < 0.0332, **p < 0.0021, ***p < 0.0002, ****p < 0.0001. Values represent mea*n* ± SEM. Scale bars indicate 50 µm in overview and 10 µm in enlarged frames. D: Day. i.p.: intraperitoneal.2 m:2-month-old.

We next aimed to understand the mechanism responsible for the therapeutic benefit afforded by FasL inhibition (Fig. 5a). Whereas treatment with mFas-Fc did not impact viral titres after infection (Fig. 5b), therapeutic inhibition of FasL significantly reduced the amount of cell death detected by TUNEL staining in the lungs of MA20-infected mice at 5 dpi as compared to Control-Fc-treated mice (Fig. 5c, d). Therapeutic inhibition of FasL significantly reduced cleaved Caspase-3 staining in the lungs of MA20-infected mice already one day after treatment (Fig. 5c, d), implying an early role for FasL-induced cell death in pathology.

We next quantified the presence of 39 soluble mediators, including cytokines and chemokines, in lung homogenates of MA20-infected *versus* mock-infected mice before and after treatment with mFas-Fc or Control-Fc (Fig. 5e and Supplementary information, Fig. S5a). As shown in the heat maps in Supplementary information, Fig. S5a, the different clusters mostly differentiated mock, control and treated mice. Analysis of these 39 factors as the cumulated inflammation driven by MA20 infection (‘Inflammation Score’) revealed that treatment with the FasL inhibitor significantly reduced the increase in the Inflammation Score in infected mice at 3 dpi (Fig. 5e). Levels of FasL were significantly increased in lung homogenates upon infection and one day after treatment with mFas-Fc, i.e. at 3 dpi, FasL levels were already decreased when compared to infected mice treated with Control-Fc (Fig. 5f).

Among others, particularly cytokines that have been shown to be upregulated in COVID-19 patients, some of them correlating with increased mortality, including CCL2, CCL3, CXCL1, CXCL10, CCL20, GM-CSF, M-CSF, IFNγ and TNF [42–45], were also decreased upon either treatment, albeit not all of them with statistical significance (Supplementary information, Fig. S5b). Interestingly, FasL blockade also resulted in a significant reduction of TNF levels in the lung homogenates of infected mice. Moreover, the most significant decrease induced by FasL inhibition was that of IFNγ (Supplementary information, Fig. S5b). In addition, other factors like the immunomodulatory calcium binding protein S100A9 as well as the tissue inhibitor of metalloproteinases-1 (TIMP-1), both of which were described to correlate with poor prognosis in COVID-19 patients [46, 47], were also significantly decreased by treatment with mFas-Fc (Supplementary information, Fig. S5b).

### FasL is increased in the bronchoalveolar lavage fluid of hospitalised COVID-19 patients

**Fig. 6:**
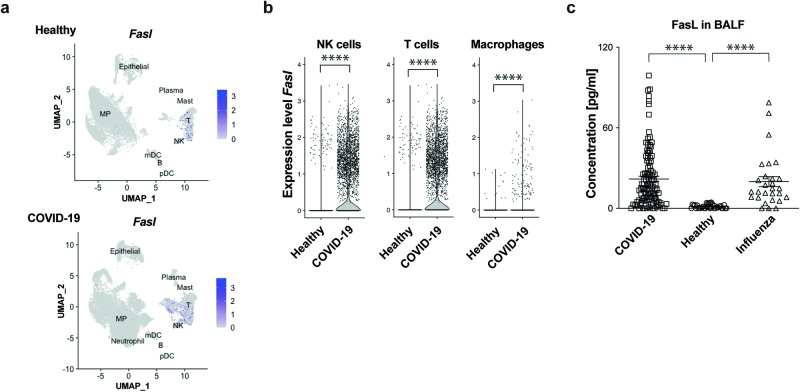
FasL upregulation in the bronchoalveolar lavage fluid of COVID-19 patients. Human BALF scRNAseq data for UMAP projection (**a**) and Violin plots (**b**) depicting *Fasl* mRNA expression in immune cell populations as indicated (NK cells, healthy *n* = 97; COVID-19 *n* = 984; T cells, healthy *n* = 1225; COVID-19 *n* = 6491; macrophages, healthy *n* = 18539; COVID-19 *n* = 30878). *p* values in Violin plots (B) were determined by Wilcoxon Rank Sum test. *p < 0.0332, **p < 0.0021, ***p < 0.0002, ****p < 0.0001. **c** Cytokine protein analysis of FasL in BALF of 24 COVID-19 ICU patients with serial sample collection during hospitalization (*n* = 109) and IAV patients (*n* = 28) and healthy donors (*n* = 36) quantified by Luminex Multiplex Assay. *p* values were determined by Mann-Whitney test. *p < 0.0332, **p < 0.0021, ***p < 0.0002, ****p < 0.0001. Values represent mea*n* ± SEM. NK cells: Natural Killer cells. BALF bronchoalveolar lavage fluid.

To determine whether the results we obtained in the MA20 mouse model could be clinically relevant for COVID-19 patients, we next analysed human scRNAseq data of cells collected the from bronchoalveolar lavage fluid (BALF) of hospitalised SARS-CoV-2-infected patients using a previously published dataset [48] (Fig. 6a, b). This revealed that *Fasl* mRNA was significantly increased in the NK cell, T cell and macrophage populations as compared to healthy donors (Fig. 6a, b). We next determined the expression of FasL at the protein level in the BALF of critically-ill COVID-19 patients who required intubation and were therefore ICU-admitted. This revealed that FasL was significantly increased in the BALF of patients who fell critically-ill due to infection by SARS-CoV-2 (Fig. 6c). Intriguingly, FasL was also significantly increased in the BALF of patients whose ICU-care-requiring critical illness was caused by Influenza A (H1N1) virus (IAV) infection (Fig. 6c). These results suggest a potential role for FasL in pathological lung damage and disease progression caused by the respiratory RNA viruses SARS-CoV-2 and IAV.

## Discussion

We here identify FasL as a crucial host factor responsible for promoting lung inflammation and disease progression in mice infected with mouse-adapted SARS-CoV-2. Importantly, therapeutic inhibition of FasL at 2dpi significantly reduced mortality, correlating with a reduction in cell death and inflammation in the lungs of MA20-infected mice. As in COVID-19 patients, in MA20-infected mice the disease severity was age-dependent and severe disease was accompanied by a dysregulated immune response characterised by lymphopenia, neutrophilia and eosinopenia as well as lung damage with oedema and hyaline membranes as prominent features of ARDS [49]. Interestingly, FasL expression was highly upregulated on IMMs and NK cells in the lungs of MA20-infected mice, immune cells previously shown to be responsible for severe lung disease following SARS-CoV infection in mice [7]. Thereby, this finding likely explains the therapeutic effect we observed when inhibiting FasL. Thus, the in-vivo results we obtained in the MA20 model, together with our observation that FasL is significantly increased in the BALF of critically-ill COVID-19 patients, provides a causal connection between FasL and severe COVID-19 and, at the same time, uncovers the translational potential of FasL blockade as a novel treatment for severe COVID-19.

It was previously shown in another mouse-adapted model of SARS-CoV-2 and in the transgenic K18-hACE2 mouse model of COVID-19 that genetic deficiency in TNF [40] and simultaneous pharmacologic inhibition of TNF and IFNγ [41], respectively, attenuated disease severity. Of note, in the MA20 model, FasL blockade resulted in the rapid and significant reduction of both cytokines, TNF and IFNγ. This result implies that FasL inhibition alone may be sufficient to effectively interfere with the pro-inflammatory signalling cascade, including the one unleashed by the combined effect of TNF and IFNγ.

Importantly, FasL neutralisation led to attenuation of disease without affecting viral titres. This indicates that FasL is responsible for inflammatory damage, but dispensable for virus control. This is in line with previous findings in other models of viral infection, as Fas–FasL deficiency did not enhance susceptibility to infection by Herpes simplex virus type 1 [50] and protected from Lymphocytic Choriomeningitis Virus-induced hepatitis without increasing susceptibility to viral infection [51]. Furthermore, patients suffering from auto-immune lymphoproliferative syndrome due to deficiency in Fas or FasL do not have an increased risk of any type of infection [52]. Thus, although it is known that cell death plays a role in viral replication and spread [53], FasL inhibition neither hampers nor enhances virus control. In summary, FasL inhibition may be both, efficacious and safe in the treatment of severe disease induced by SARS-CoV-2 infection. Given this clinical potential, we recently tested asunercept (Fas-Fc/CD95-Fc), the human homologue of the mFas-Fc protein we employed in this study, in a randomised, controlled clinical phase 2 trial in hospitalised COVID-19 patients requiring oxygen supplementation [54].

Intriguingly, FasL was not only significantly increased in the BALF of critically-ill patients when severe disease was caused by SARS-CoV-2 but also when caused by IAV. It is tempting to speculate that therapeutic inhibition of FasL may also provide benefit to patients developing severe disease as a consequence of infection by other respiratory RNA viruses, including those with pandemic-causing potential that may emerge in the human population in the future [55].

## Methods

### Mice and cell lines

Female BALB/c (2-month-old) and female C57BL/6J (termed C57BL/6 within the manuscript) mice (2-, 5-, 8-, 11-month-old) were purchased from Envigo. For in vivo infection experiments, mice were maintained in Biosafety Level 3 laboratories (BSL3) in Zaragoza, with *ad libitum* food and water, and under standard conditions of temperature, humidity and dark/light cycle, following the Federation for Laboratory Animal Science Associations (FELASA) directions. Protocols and animal experimentation was approved by the Animal Experimentation Ethics Committee of the University of Zaragoza (number: PI55/21).

Vero E6 cells were kindly provided by Júlia Vergara from the Centro de Investigación en Sanidad Animal IRTA-CReSA (Barcelona, Spain). Vero E6 cells were grown in DMEM (Sigma) supplemented with 10% FBS (Sigma), 2 mM Glutamax (Gibco), 100 U/ml penicillin (Sigma), 100 μg/ml streptomycin (Sigma), 0.25 μg/ml amphotericin B (Sigma), 1% non-essential amino acids (Gibco) and 25 mM HEPES (4-(2-hydroxyethyl)-1-piperanzineethanesulfonic acid) (Biowest).

### Virus expansion and titre determination by TCID50

The SARS-CoV-2, hCoV-19/Sweden/20-53846/2020, B.1.1.7. variant (Alpha) has been provided by The Public Health Agency of Sweden to improve the quality of diagnostics relevant for infectious disease control, treatment and/or other studies of relevance for public health.

The Alpha variant was expanded in Vero E6 cells to obtain high-titre batches. Virus concentration in batches was determined by TCID50 in Vero E6 cells with serial 1-log dilutions, calculated using Ramakrishan newly proposed method formula 72 h after cell infection [56] and normalized to weight (g) of lung and amount of buffer (ml) used for lysis of cells. Virus titration in lungs required previous homogenization of weighed lung pieces in 500 µl DMEM with a GentleMACS Dissociator (Miltenyi), centrifugation at 1500 rpm for 5 min and the supernatant was taken for titration. All procedures involving infectious virus, including in vivo experiments, were performed under biosafety level 3 (BSL3) conditions.

### Serial in vivo passaging of virus in mice

Serial passaging of SARS-CoV-2 Alpha in mice was performed in 1-year-old C57BL/6J male mice. Briefly, 2-3 mice were inoculated with 40 µl of the virus intranasally. At 2–3 days post infection (dpi), mice were euthanized and lungs were pooled, homogenized in DMEM, clarified by centrifugation (1500 rpm × 5 min) and used to infect naïve mice. In passages 5, 10, 15 and 20, virus was isolated. Clones from passage 20 were isolated by plaque assay using Vero E6 cells. Passages 5, 10, 15 and clones of passage 20 were utilized for deep sequencing. Clone #4 was chosen for expansion and further study due to its lethality, termed SARS-CoV-2 MA20.

### Viral whole-genome sequencing

Nucleotide acids from the initial SARS-CoV-2 Alpha variant as well as the passages P5, P10, P15 and final MA20 passage were purified from Swabs or lung homogenates with MagnNA Pure 96 system using DNA and Viral NA Large Volume Kit (Roche). SARS-CoV-2 whole genome sequences were generated using LunaScript RT SuperMix kit (NEB) for cDNA synthesis followed by the EasySeq™ SARS-CoV-2 WGS kit (Nimagen) and sequenced on Illumina NextSeq 500 or NextSeq 2000. Illumina data was processed with SeqIT’s internal “deeptypeHIV” bioinformatics pipeline specifically designed for viral resistance analysis from next generation sequencing data. The pipeline is validated for routine clinical diagnostics and can be used for different viruses. Briefly, sequence data is first mapped onto a given reference. From this mapping, a consensus sequence is generated and used as the new reference. The process is repeated several times for better analysis of samples that are distant to the original reference and to better account for large variable loops and insertions/deletions. In a separate step, the frequencies of nucleotides (and codons/amino acids) at the different positions are counted. These frequencies are available irrespective a possible coverage threshold. However, for generation of final consensus sequence at different minority cut-offs, a coverage threshold of 30 was used.

Sequence alterations were analysed with Nextclade (https://clades.nextstrain.org/) by comparing the acquired sequences to SARS-CoV-2 Wuhan strain.

### MD simulations

The starting coordinates for the complex between SARS-CoV-2 RBD bound to human ACE2 receptor were taken from RCSB (PDB ID: 6MJ0) [34]. The structures of mouse ACE2 and mutated MA20 were obtained by constructing the corresponding homology models using the Schrodinger package (Schrödinger Release 2023-1: Desmond Molecular Dynamics System, D. E. Shaw Research, New York, NY, 2021. Maestro-Desmond Interoperability Tools, Schrödinger, New York, NY, 2021). The mutated MA20 was located by superimposition to the RBD obtained from the crystal structure. Similarly, mouse ACE2 was obtained by mutagenesis in silico of human ACE2 obtained from the crystal structure. Force field parameters for the sugar moieties were taken from GLYCAM06_H parameters for the GlNAc unit. Each complex was immersed in a truncated octahedral box with a 12 Å buffer of TIP3P water molecules and neutralized by adding explicit counter ions (Na+, Cl−). All subsequent simulations were performed using the ff19SB force field [57]. A two-stage geometry optimization approach was used in the framework of AMBER22 [58] package of programs. The first stage minimizes only the positions of solvent molecules and ions, and the second stage is an unrestrained minimization of all the atoms in the simulation cell. The systems were then gently heated by incrementing the temperature from 0 to 300 K under a constant pressure of 1 atm and periodic boundary conditions. Harmonic restraints of 50 kcal/mol were applied to the solute, and the Andersen temperature coupling scheme was used to control and equalize the temperature. The time step was kept at 1 fs during the heating stages. Water molecules are treated with the SHAKE algorithm such that the angle between the hydrogen atoms is kept fixed. Long-range electrostatic effects are modelled using the particle-mesh-Ewald method. An 8-Å cut-off was applied to Lennard–Jones and electrostatic interactions. Each system was equilibrated for 2 ns with a 2-fs time step at a constant volume and temperature of 300 K. Production trajectories were then run for additional 500 ns under the same simulation conditions.

### Cloning and production of SARS-CoV-2 spike and *mus musculus* ACE2 (mACE2) proteins

The following coronavirus regions were amplified from synthetic gene plasmids or cDNA and cloned into modified Sleeping Beauty transposon expression vectors [59]: CoV-2 Spike Alpha N-term: (MN908947; AA: 13–535; V70I, Y145H, N501Y, N-terminal BM40 – Twin-Strep-tag; 65 kDa); CoV-2 Spike MA20 N-term: (MN908947; AA: 13-535; del69-70, Y145H, Q493R, N501Y, Y505H, N-terminal BM40 – Twin-Strep-tag; 65 kDa); mACE-2 ecto: (NP_001123985; AA: 20–611; 5′-BM40 signal peptide and a 3′-8 × histidine tag). For recombinant protein production, stable HEK293 EBNA cell lines were generated using the Sleeping Beauty transposon system [59]. Expression constructs, after verifying by sequencing, were co-transfected with a transposase plasmid (10:1) into the HEK293 EBNA cells using FuGENE® HD transfection reagent (Promega GmbH, Madison, USA) in DMEM/F12 supplemented with 6% FBS. After high puromycin selection (3 µg/ml; Sigma), cells were expanded in triple flasks and protein production induced with doxycycline (0.5 µg/ml, Sigma). Supernatants of confluent cells were harvested every 3 days, filtered and recombinant proteins purified via Strep-Tactin®XT (IBA Lifescience, Göttingen, Germany) resin. After a 1 M NaCl wash step, proteins were eluted with biotin containing buffer (IBA Lifescience, Göttingen, Germany). In the case of mACE-2 ecto, serum free supernatant was filtered and applied to Ni-NTA Agarose (PureCube 100 INDIGO Ni-Agarose; Cube Biotech, Monheim, Germany). After washing the column with increasing concentration of imidazole, mACE-2 ecto was eluted with 200 mM imidazole in TBS (pH 8.0). The proteins were dialyzed against TBS, analysed by SDS-PAGE (PageBlue™ Protein Staining Solution; Thermo Fisher Scientific), and stored at 4 °C or −80 °C.

### ELISA style binding assay

For the binding assay, mACE2 was diluted in TBS (pH 8.0) to 10 μg/ml and 0.5 µg/well (50 µl/well) were coated (96-well plates, Nunc Maxisorb) at 4 °C overnight. After washing with TBS, unspecific binding sites were blocked at room temperature with 100 µl 2% BSA in TBS for 1.5 h. S1 Spike proteins (Strep II tagged) were then serial diluted in blocking buffer from 2 µM to 2 nM in 50 µL per well. After 1.5 h incubation at room temperature the ELISA plates were carefully washed 3 times with TBS buffer and Strep-Tactin® HRP conjugate 1:3000 (IBA-Lifesciences, Göttingen, Germany) in blocking buffer was applied for 1 h at room temperature. Afterwards the plates were washed 3 times. Horseradish peroxidase activity was detected utilizing the 1-Step Ultra TMB ELISA substrate solution (ThermoFisher Scientific). The reaction was stopped after around 5 min by adding 50 µl 10% H_2_SO_4_ and the absorbance was measured at 450 nm. The evaluation was done with GraphPad Prism 5. The apparent Kd from three measurements of the MA20 binding to mACE2 is 116 ± 21.5 nM.

### In vivo infection and disease monitoring

Mice were anaesthetized with isoflurane and infected intranasally with the indicated amount of virus in a total volume of 40 µl PBS. Mice were weighed and a clinical score was performed during the time of experiment. The clinical score followed different components: mouse appearance, level of consciousness, activity, response to stimuli, eye appearance and frequency and quality of respiration, standardized to a five-point scale ranging from 0–4 [60]. Mice reached humanitarian end point with a 25% weight loss, when the clinical score reached 15 or if any of the respiratory characteristics increased by more than 3. Animal experimentation was approved by the Animal Experimentation Ethics Committee of the University of Zaragoza (number: PI55/21).

### Histology and immunohistochemistry

Upon euthanasia, organs were harvested, washed in sterile PBS before fixation with formaldehyde 3.7–4% w/v buffered to pH 7 and stabilised with methanol (PanReac AppliChem, 252931) for 24 h at 4 °C. The following day organs were embedded in paraffin. Tissue samples in paraffin blocks were cut with the microtome (4 µm) and stained for haematoxylin-eosin staining, SARS-CoV N protein immunohistochemistry and TUNEL staining.

For the haematoxylin-eosin staining, briefly, paraffin was removed by xylene and alcohol immersion before the haematoxylin and eosin stainings.

Tissues were evaluated following the ALI score [61].

For the ASL/ALI scoring system the following parameter were analysed:

A. neutrophils in the alveolar space (none = 0, 1-5 cells = 1, > 5 cells = 2);
B. neutrophils in the interstitial space/septae (none = 0, 1-5 cells = 1, > 5 cells = 2);
C. hyaline membranes (none = 0, one membrane = 1; > 1 membrane = 2);
D. proteinaceous debris in air spaces (none = 0, one instance = 1, > 1 instance = 2);
E. alveolar septal thickening ( > 2x mock thickness = 0, 2-4x mock thickness = 1, > 4xmock thickness = 2).

Scores were calculated as followed: [(20 × A) + (14 × B) + (7 × C) + (7 × D) + (2 × E)]/100.

Final scores were obtained by averaging three fields per mouse.

For SARS-CoV-2 antigen detection, the paraffin from the slides was initially removed by xylene and alcohol. For epitope retrieval samples were hydrated autoclaved in PT-Link (DAKO) with Tris-EDTA buffer for 20 min at 96 °C, and for immunohistochemistry staining samples were incubated with blocking reagent (PBS with 10% normal goat serum for 30 min) followed by rabbit monoclonal antibody against SARS-CoV-2 N protein (1:20,000 dilution for 60 min, number 40143-R019, Sino Biological), and then incubated with rabbit Envision (Dako) and diaminobenzidine (Dako) as chromogen and counterstained with haematoxylin.

Immunohistochemical analysis of cleaved caspase 3 was performed using an enzymatic, non-biotin amplification system, the ImmPRESS® HRP Goat Anti-Rabbit IgG Polymer Detection Kit, Peroxidase (MP-7451) (Vector Laboratories, Burlingame, CA, USA). The mouse monoclonal antibody specifically recognising the Asp175 residue of CC3 (diluted 1:200 overnight at 4 °C) (Cell Signalling Technology, Danvers, MA, USA) was used after heat-mediated antigen retrieval with pH 6.0 citrate buffer.

Image acquisition was carried out using a NanoZoomer 2.0-HT slide scanner (Hamamatsu, Bridgewater, NJ, USA) and analysis and quantification were done using QuPath Positive cell detection command in in five representative regions of 500 ×500 µm per section.

### TUNEL assay

Cell death in FFPE lung tissue was detected resourcing to a TUNEL Assay kit - ApopTag® Red In situ Apoptosis Detection Kit (Millipore, Billerica, MA, USA, S7165). Staining was performed according to the manufacturer protocol. Briefly, sections were deparaffinised and hydrated followed by antigen retrieval with Proteinase K. Sections were then submitted to an incubation with equilibration buffer for 30 s followed by 1 h with TdT reaction mixture at 37 °C. Anti-digoxigenin antibody conjugated with a rhodamine fluorochrome was incubated for 30 min followed by 20 min incubation with DAPI for nuclear contrast.

Image acquisition was done using a Leica Stelaris 5 Confocal Microscope with a 20× and 40× lens. Image analysis and quantification were done using the Voronoi Threshold Labeler tool from the BioVoxxel Toolbox [62] by analysis of five representative regions of 500 × 500 µm per section. The number of TUNEL-positive objects was normalized to number of cells (DAPI-positive objects) (Fig. S4a).

### Lung cell isolation and cell staining for flow cytometry analysis

Animals were euthanized and lungs were harvested, homogenated and incubated with Liberase TM and DNAseI for 30 min at 37 °C before further disaggregation. Single cell suspensions were prepared by passaging lung tissue through a 70 µm cell strainer. For viability staining Viobility was used at 1 µl per sample in 20 µl PBS, for 10 min at room temperature. Afterwards, respective antibodies were added and incubated for 20 min at 4 °C. Cells were washed with PBS and fixed with PFA 4% before flow cytometry using a Gallios (Beckman Coulter) and analysed with Kaluza Software (Beckman Coulter). Antibodies used for different panels are shown in Table S1. The gating strategy is shown in Fig. S2.

### RNA isolation and total RNAseq

For RNAseq analysis a piece of lung upon euthanasia was stored in RNAprotect(Qiagen) and further processed following RNAeasy kit (Qiagen) according to manufacturer’s instructions. Purified RNA was quantified by spectrophotometry using a NanoDrop (NanoDrop Technologies, USA.

Total RNA Sequencing was performed by CeGaT GmbH (Tübingen, Germany). In brief, 10 ng RNA of each sample were used for library preparation with the SMART-Seq stranded total RNA kit (Takara). Libraries were sequenced on a NovaSeq 6000 machine (Illumina) with 2 × 100 bp.

The sequencing reads were demultiplexed with Illumina bcl2fastq (2.20) and adaptors were trimmed with Skewer (version 0.2.2) [63]. Quality trimming of the reads has not been performed. Trimmed raw reads were aligned to the mouse reference genome (mm10) or for viral analyses to SARS CoV2 (MN908947) reference genome using STAR (version 2.7.3) [64]. The raw counts derived from the mapping contain the number of reads that map to each gene ID. Based on these numbers the normalized counts were calculated. Normalized counts have been calculated with DESeq2 (version 1.24.0) [65] in R (version 3.6.1) (R Core Team 2015). Genes with less than two reads over all samples had been removed from analysis. The quality of the FASTQ files was analysed with FastQC (version 0.11.5-cegat) [66].

Pathway enrichment analysis of differentially expressed genes at day 2 was performed using the pathfind R package. Gene expression comparisons between control/mock and different days of various treatment were performed using the DESeq2 R package [65].

### scRNAseq analysis

Published data set from Liao et al. was used for scRNAseq data analysis [48]. MAST in Seurat v.3 (FindMarkers function) was used to perform differential gene expression analysis. Cell populations were defined based on the expression of the respective markers as described in Liao et al. [48]. After selecting the respective cell populations, we compared expression differences between samples from COVID-19 patients and healthy donors for *Fasl* expression. For macrophages, T and NK cells, DEGs were generated comparing COVID-19 *versus* healthy donors for the expression of the *Fasl* gene. We identified significantly different expression between two groups of cells using Wilcoxon Rank Sum test (wilcox.test in FindMarkers function, default test). Violin plots were drawn by VlnPlot function in Seurat of single cell data comparing gene expression between COVID-19 and healthy donors.

### Cytokine and chemokine analysis

Lung samples were obtained from a weighed portion of murine lung, previously homogenized in 500 µl DMEM with a GentleMACS Dissociator (Miltenyi), and clarified, taking the supernatant after centrifugation (1500 rpm × 5 min) for analysis. Prior to analysis virus was inactivated by addition of 0.5% Triton X100 to the homogenate and incubated for 30 min at 4 °C. Protease inhibitor (Complete, Roche) was added to prevent degradation during incubation.

Respiratory tract fluid samples of COVID-19 and Influenza A patients were sampled by endotracheal aspiration in intubated patients at the Intensive Care Unit of the Medical University of Vienna and the Klinik Favoriten in Vienna, Austria, as part of the Austrian CoronaVirus Adaptive Clinical Trial (ACOVACT; NCT04351724, Ethics #1315/2020). Further information of individual patients for SARS-CoV-2 copy number and time of sample collection is listed in Table S2. We further analysed bronchoalveolar lavage fluid (BALF) samples from a previous study on a human endotoxemia model; detailed methods have been described before [67]. In short: twenty-four healthy volunteers received either 2 × 40 mg intravenous dexamethasone or placebo and had endotoxin instilled into a lung segment and saline instilled into a contralateral segment (serving as control). A bilateral BAL was performed 6 h after endotoxin instillation. Only samples with saline instillation have been utilized for analysis of healthy controls. No filtration has been performed prior to storage of the samples at −80 °C.

Cytokine array was performed *via* Luminex Discovery Assay (R&D Biotechne) with the indicated analytes according to the manufacturer’s instructions. Samples were centrifuged at 1000 × *g* for 10 min and diluted 1:4 (human) or 1:2 (mouse) in Calibrator Diluent (R&D Biotechne) prior to analysis. In addition, in BALF samples virus was inactivated by additional incubation with 4% FA for 1 h at 800 rpm at RT as last step. Cytokines/Chemokines were measured with Luminex 200 xMAP system (Luminex) and quantified by comparison to a standard curve. xPONENT software was used for data collection and analysis.

Cytokine and chemokine murine lung samples measured by Luminex were clustered by agglomerative hierarchical clustering based on the levels of cytokines. First, samples distances were computed via the R function dist, with Euclidean distance. Next, hclust function generated a clustering from the distances, with “Ward-D” linkage method. The same process was performed for cytokine distances. Finally, for visualization purposes, a heatmap was plotted with the representation of the 44 samples and 39 cytokines levels, previously transformed to log2, scaled and centred. A dendrogram was drawn to visualize the distance tree for samples and cytokines. To note, values that did not match Luminex quality criteria and outliers were settled to “0” and marked as blank grey squares in the heatmap. BALF samples have been stored and processed under BSL2 conditions.

### Fc-protein production

The murine Fas-Fc protein was designed by fusing the extracellular domain of the receptor to the Fc portion of murine IgG2a (mIgG2a) and, like the Control-IgG, was produced by WuXi Biologics. In brief, CHO-K1 cells were transfected with the respective expression construct and cell culture supernatants were collected for purification to gain at least 98% purity and endotoxin levels <0.1 EU/mg. Proteins were formulated in histidine buffer with 20 mM histidine and 150 mM NaCl, pH 5.5. Quality control was performed via A280, SDS-PAGE and SEC-HPLC. The mFas-Fc sequence is provided in Table S3.

### In vivo treatments

Recombinant mFas-Fc-protein was tested in vitro for activity prior to in vivo experiments. Treatments were administered intraperitoneally with 500 µg in 200 µl PBS to mice at 2 dpi in blind and randomised groups.

### Statistics and reproducibility

Please refer to the legend of the figures for description of sample size (n) and statistical significance. If not stated otherwise, data were analysed with GraphPad Prism 9 software using the statistical tests indicated in the respective figure legends. ns: not significant.

### Supplementary information

Supplemental Material

Figure S1

Figure S2

Figure S3

Figure S4

Figure S5

## Data Availability

Further information and requests for resources and reagents should be directed to and will be fulfilled by the Lead Contact. The raw RNA-sequencing data generated in this study is in the process of being deposited at the Gene Expression Omnibus, and the accession number will be provided, as any other data produced in the experiments, upon reasonable request.
