# Supplementary material for: Identification of FasL as a crucial host factor driving COVID-19 pathology and lethality
Source: Cell Death Differ. 2024 Mar 21;31(5):544–57. doi: 10.1038/s41418-024-01278-6 (PMC11093991; doi:10.1038/s41418-024-01278-6)
Supplement: Supplementary file 1 — Supplemental Material [file 41418_2024_1278_MOESM1_ESM.docx]

**Supplementary Figures**

**Fig. S1. MA20-caused disease severity is titre- and age-dependent.**

(**a**) Viral titres of the intermediate passages during SARS-CoV-2 adaptation.

(**b**) Weight loss curves of 12m C57BL/6 mice infected with Passage 7 of SARS-CoV-2 adaptation (n=3).

(**c**)Weight loss curves of titration of MA20 with increasing age of C57BL/6 mice and increasing concentration of MA20 virus (n = 4). Black dots indicate when mice reached humane endpoint.

(**d**) Weight loss curves with increasing concentrations of MA20 virus in 2m BALB/c mice (n = 4 to 6).

(**e**) Survival curves of 2m BALB/c mice infected with indicated viral titres (n = 4 to 6).

(**f-i**) Survival curves of 2m C57BL/6 (**f**), 5m C57BL/6 (**g**), 8m C57BL/6 (**h**) and 11m C57BL/6 (**i**) mice infected with indicated viral titres of MA20 (n = 4).

D: Day. 2m: 2-month-old. 5m: 5-month-old. 8m: 8-month-old. 11m: 11-month-old.

**Fig. S2. Acquired mutations in MA20 enhance binding affinity to mACE2.**

(**a**)RMSD values (nm) of the backbones of the indicated constructs during 500 ns MD simulations of chains A (hACE2 or mACE2, green) and E (Alpha or MA20, blue).

(**b**) Distances (Å) corresponding to the observed interactions along 500 ns of MD simulations between atoms of key residues of chains A (hACE2 or mACE2) and E (RBD of Alpha or MA20). The distances from the carboxyl groups correspond to the distance from the centre of mass of the carboxyl group (O-C-O atoms).

(**c**) Detail of the distances (Å) corresponding to the observed interactions along 500 ns of MD simulations between D38 and Y449. Red and black traces correspond to the distances between the two indistinguishable oxygen atoms (OD1 and OD2) of the carboxyl group of the side chain of D38 and the phenolic oxygen of Y449.

(**d**) Coomassie-Gel of amino acid (AA) sections of recombinant Spike and mACE2 proteins utilized for ELISA-style binding assay.

(**e**) IHC staining of brain sections of SARS-CoV-2 Nucleocapsid protein of infected 2m BALB/c, 2m C57BL/6 and 8m C57BL/6 mice.

Scale bars indicate 500 µm. AA: Amino acids. 2m: 2-month-old. 8m: 8-month-old.

**Fig. S3. MA20 promotes immune cell dysregulation.**

**(a** and **b)** Lung weight of 2m BALB/c (a), 2m C57BL/6 and 8m C57BL/6 (b) mice after MA20 infection (n = 4 to 6). *p* values were determined by One-way ANOVA with post-hoc Tukey. *p < 0.0332, **p < 0.0021, ***p < 0.0002, ****p < 0.0001.

**(c** and **d)** Gating strategy of myeloid flow cytometry panel (c) and lymphoid flow cytometry panel (d) as indicated.

**(e** and **f)** Flow cytometry analysis of immune cell populations in the lungs of infected 2m BALB/c (e), 2m C57BL/6 and 8m C57BL/6 (f) mice as indicated (n = 3 to 6). *p* values were determined by One-way ANOVA with post-hoc Tukey. *p < 0.0332, **p < 0.0021, ***p < 0.0002, ****p < 0.0001.

**(g** and **h)** ELR of infected 2m BALB/c (g), 2m C57BL/6 and 8m C57BL/6 (h) mice (n = 3 to 6). *p* values were determined by One-way ANOVA with post-hoc Tukey. *p < 0.0332, **p < 0.0021, ***p < 0.0002, ****p < 0.0001.

Values represent mean ± SEM. NK cells: Natural Killer cells. ELR: Eosinophil / Lymphocyte ratio. 2m: 2-month-old. 8m: 8-month-old.

**Fig. S4. Cell death after MA20 infection within the lungs of infected mice.**

**(a)** PC cluster analysis of bulk RNAseq of infected lungs from 2m BALB/c mice at indicated days (n = 4 to 6).

**(b)** Example of representative image selection for TUNEL-stained lung sections of infected 2m BALB/c mice.

**(c)** Flow cytometry expression analysis of control staining for FasL expression on immune cells as indicated.

Scale bars indicate 1000 µm in overview (a, left) and 100 µm in enlarged frames (a, right). FMO: Fluorescence-minus-one (control). Inflamm. Mono-Macs: Inflammatory Monocytic-Macrophages. NKs: Natural Killer cells. 2m: 2-month-old.

**Fig. S5. FasL inhibition decreases soluble proteins correlating with COVID-19 severity.**

**(a)** Heatmap of Luminex Multiplex Assay with hierarchical clustering grouping samples based on levels of cytokine and chemokine expression, log transformed (n = 9 or 5). Dendrograms was drawn for distance-tree visualization purposes. Coloured bars on top represent respective treatments and days as indicated after infection of 2m BALB/c mice.

**(b)** Individual protein levels of (left to right) CCL2, CCL3, CCL20, CXCL1 (upper panels), CXCL10, S100A9, GM-CSF, M-CSF (middle panels) and TIMP-1, IFNγ and TNF (lower panel) in infected lung homogenates of 2m BALB/c mice with indicated treatment (n = 9 or 5). *p* values were determined by One-way ANOVA with post-hoc Dunnett. *p < 0.0332, **p < 0.0021, ***p < 0.0002, ****p < 0.0001.

Values represent mean ± SEM. 2m: 2-month-old.

**Supplementary Tables**

| **Panel** | **Antibody** | **Fluoro-chrome** | **Channel Gallios** | **Clone** | **Reference** | **Company** |
| --- | --- | --- | --- | --- | --- | --- |
| Myeloid | Viability Dye Green | 488 | FL1 |  | 130-110-207 | Miltenyi |
|  | SiglecF | PE | FL2 | E50-2440 | 562068 | BD Bioscience |
|  | MHC-II | PE-Dazzle (CF594) | FL3 | M5/114.15.2 | 107647 | Biolegend |
|  | Ly6G | BB700 | FL4 | 1A8 | 566453 | BD Bioscience |
|  | Ly6C | PE-Cy7 | FL5 | HK1.4 | 128017 | Biolegend |
|  | CD11b | APC | FL6 | M1/70 | 101212 | Biolegend |
|  | CD11c | Alexa Fluor 700 | FL7 | N418 | 117320 | Biolegend |
|  | CD64 | APC-Fire750 | FL8 | X54-5/7.1 | 139333 | Biolegend |
|  | FasL | Biotin + Strep-BV421 | FL9 | MFL3 | 106603 + 405225 | Biolegend |
|  | CD45 | VioGreen | FL10 | 30F11 | 130-123-900 | Miltenyi |
| Lymphoid | Viability Dye Green | 488 | FL1 |  | 130-110-207 | Miltenyi |
|  | NKp46 | PE | FL2 | 29A1.4 | 137604 | Biolegend |
|  | CD62L | PE-Dazzle (CF594) | FL3 | DX5 | 130-123-702 | Miltenyi |
|  | CD4 | BB700 | FL4 | GK1.5 | 745922 | BD Bioscience |
|  | CD8a | PE-Cy7 | FL5 | 53-6.7 | 100721 | Biolegend |
|  | CD44 | APC | FL6 | IM7 | 103012 | Biolegend |
|  | CD11c | Alexa Fluor 700 | FL7 | N418 | 117320 | Biolegend |
|  | CD3 | APC-Fire750 | FL8 | 17A2 | 100247 | Biolegend |
|  | FasL | Biotin + Strep-BV421 | FL9 | MFL3 | 106603 + 405225 | Biolegend |
|  | CD45 | VioGreen | FL10 | 30F11 | 130-123-900 | Miltenyi |

**Table S1**: Antibody list used for FACS panels, related to flow cytometry analysis.

| Patient ID | SARS-CoV-2 copies | days since intubation | death |
| --- | --- | --- | --- |
| SARS-FP-1 | 66000000 | 0 | no |
| SARS-FP-1 | 15000 | 2 | no |
| SARS-FP-1 | 66000000 | 7 | no |
| SARS-FP-1 | 110000 | 19 | no |
| SARS-FP-87 | 2000000 | 1 | no |
| SARS-FP-87 | 250000 | 4 | no |
| SARS-FP-87 | 3800 | 8 | no |
| SARS-FP-87 | 250000 | 12 | no |
| SARS-FP-53 | 120000 | 6 | no |
| SARS-FP-53 | 7600 | 9 | no |
| SARS-FP-53 | 500 | 11 | no |
| SARS-FP-53 | 500 | 13 | no |
| SARS-FP-4 | 2000000 | 2 | 25.04.20 |
| SARS-FP-4 | 930 | 11 | 25.04.20 |
| SARS-FP-4 | 500 | 16 | 25.04.20 |
| SARS-FP-4 | 500 | 18 | 25.04.20 |
| SARS-FP-4 | 500 | 21 | 25.04.20 |
| SARS-FP-88 | 120000 | 1 | no |
| SARS-FP-88 | 250000 | 4 | no |
| SARS-FP-88 | 31000 | 8 | no |
| SARS-FP-31 | 4100000 | 0 | no |
| SARS-FP-31 | 500000 | 9 | no |
| SARS-FP-31 | 250000 | 12 | no |
| SARS-FP-31 | 62000 | 14 | no |
| SARS-FP-31 | 500 | 16 | no |
| SARS-Resp-2 | 110000 | 3 | no |
| SARS-Resp-2 | 15000 | 6 | no |
| SARS-Resp-2 | 500 | 9 | no |
| SARS-Resp-2 | 100 | 14 | no |
| SARS-Resp-2 | 100 | 20 | no |
| SARS-Resp-2 | 100 | 23 | no |
| SARS-Resp-2 | 100 | 27 | no |
| SARS-Resp-2 | 100 | 29 | no |
| SARS-Resp-2 | 100 | 30 | no |
| SARS-FP-56 | 110000 | 3 | 22.05.20 |
| SARS-FP-56 | 790000 | 6 | 22.05.20 |
| SARS-FP-56 | 1900 | 27 | 22.05.20 |
| SARS-FP-90 | 31000 | 3 | 03.04.20 |
| SARS-FP-90 | 16000000 | 6 | 03.04.20 |
| SARS-FP-90 | 66000000 | 8 | 03.04.20 |
| SARS-FP-90 | 16000000 | 10 | 03.04.20 |
| SARS-FP-91 | 4000000 | 7 | 20.04.20 |
| SARS-FP-91 | 33000000 | 12 | 20.04.20 |
| SARS-FP-91 | 210000 | 14 | 20.04.20 |
| SARS-FP-92 | 16000000 | 1 | no |
| SARS-FP-92 | 100 | 8 | no |
| SARS-FP-92 | 500 | 13 | no |
| SARS-FP-92 | 100 | 17 | no |
| SARS-FP-93 | 530000000 | 0 | no |
| SARS-FP-93 | 66000000 | 4 | no |
| SARS-FP-93 | 4000000 | 14 | no |
| SARS-FP-93 | 2000000 | 18 | no |
| SARS-FP-93 | 31000 | 19 | no |
| SARS-FP-93 | 500000 | 22 | no |
| SARS-FP-93 | 120000 | 30 | no |
| SARS-FP-93 | 2100 | 32 | no |
| SARS-FP-93 | 560 | 35 | no |
| SARS-FP-94 | 530000000 | 5 | 10.04.20 |
| SARS-FP-94 | 16000000 | 8 | 10.04.20 |
| SARS-Resp-1 | 3000000 | 1 | no |
| SARS-Resp-1 | 410000 | 7 | no |
| SARS-Resp-1 | 210000 | 8 | no |
| SARS-Resp-1 | 6000 | 12 | no |
| SARS-Resp-1 | 500 | 18 | no |
| SARS-Resp-1 | 100 | 21 | no |
| SARS-Resp-1 | 100 | 25 | no |
| SARS-Resp-1 | 100 | 25 | no |
| SARS-Resp-1 | 100 | 27 | no |
| SARS-Resp-1 | 100 | 28 | no |
| SARS-Resp-3 | 100 | 39 |  |
| SARS-Resp-3 | 100 | 40 |  |
| SARS-Resp-3 | 100 | 42 |  |
| SARS-Resp-3 | 100 | 43 |  |
| SARS-FP-3 | 250000 | 2 | no |
| SARS-FP-3 | 1000000 | 7 | no |
| SARS-FP-3 | 7800 | 9 | no |
| SARS-FP-3 | 4000 | 12 | no |
| SARS-FP-3 | 1100 | 16 | no |
| SARS-FP-3 | 100 | 19 | no |
| SARS-FP-3 | 100 | 21 | no |
| SARS-FP-42 | 16000000 | 4 | no |
| SARS-FP-42 | 500000 | 7 | no |
| SARS-FP-42 | 62000 | 9 | no |
| SARS-FP-42 | 15000 | 11 | no |
| SARS-FP-95 | 16000000 | 0 | 27.03.20 |
| SARS-FP-95 | 1000000 | 1 | 27.03.20 |
| SARS-FP-95 | 500000 | 2 | 27.03.20 |
| SARS-Resp-4 | 500000 | 1 | no |
| SARS-Resp-4 | 1900 | 5 | no |
| SARS-Resp-4 | 100 | 5 | no |
| SARS-Resp-4 | 500 | 7 | no |
| SARS-Resp-4 | 100 | 10 | no |
| SARS-Resp-4 | 100 | 12 | no |
| SARS-Resp-4 | 100 | 13 | no |
| SARS-Resp-4 | 100 | 14 | no |
| SARS-FP-96 | 61000 | 5 | no |
| SARS-FP-96 | 8100000 | 9 | no |
| SARS-FP-96 | 500 | 28 | no |
| SARS-FP-97 | 2000000 | 1 | no |
| SARS-FP-97 | 120000 | 3 | no |
| SARS-FP-98 | 270000000 | 3 | 30.04.20 |
| SARS-FP-98 | 270000000 | 7 | 30.04.20 |
| SARS-FP-98 | 130000000 | 10 | 30.04.20 |
| SARS-FP-98 | 33000000 | 14 | 30.04.20 |
| SARS-FP-98 | 33000000 | 17 | 30.04.20 |
| SARS-FP-98 | 4000000 | 19 | 30.04.20 |
| SARS-FP-98 | 15000 | 21 | 30.04.20 |
| SARS-FP-98 | 930 | 25 | 30.04.20 |
| SARS-FP-98 | 2100 | 35 | 30.04.20 |
| SARS-FP-98 | 15000 | 38 | 30.04.20 |
| SARS-FP-98 | 500 | 45 | 30.04.20 |
| SARS-FP-99 | 2000000 | 2 | no |
| SARS-FP-99 | 500000 | 6 | no |
| SARS-FP-99 | 500 | 14 | no |
| SARS-FP-99 | 100 | 21 | no |
| SARS-FP-100 | 1000000 | 1 | 14.04.20 |
| SARS-FP-100 | 120000 | 4 | 14.04.20 |
| SARS-FP-100 | 61000 | 6 | 14.04.20 |
| SARS-FP-100 | 2000000 | 8 | 14.04.20 |
| SARS-FP-100 | 1000000 | 13 | 14.04.20 |
| SARS-FP-100 | 61000 | 15 | 14.04.20 |
| SARS-FP-100 | 3800 | 18 | 14.04.20 |

**Table S2**: Clinical parameters and collection day of patients for BALF analysis; value 100 of SARS-CoV-2 copies is equivalent to no virus detectable, related to Luminex analysis.

| **Name** | Signalling peptide – **Extracellular Domain** – mIgG2a |
| --- | --- |
| mFas-Fc | MGWSCIILFLVATATGVHS**QGTNSISESLKLRRRVRETDKNCSEG**  **LYQGGPFCCQPCQPGKKKVEDCKMNGGTPTCAPCTEGKEYMD**  **KNHYADKCRRCTLCDEEHGLEVETNCTLTQNTKCKCKPDFYCD**  **SPGCEHCVRCASCEHGTLEPCTATSNTNCRKQSPRN**GPTIKPC  PPCKCPAPNLLGGPSVFIFPPKIKDVLMISLSPIVTCVVVDVSEDD  PDVQISWFVNNVEVHTAQTQTHREDYNSTLRVVSALPIQHQDW  MSGKEFKCKVNNKDLPAPIERTISKPKGSVRAPQVYVLPPPEEE  MTKKQVTLTCMVTDFMPEDIYVEWTNNGKTELNYKNTEPVLDS  DGSYFMYSKLRVEKKNWVERNSYSCSVVHEGLHNHHTTKSFS  RTPGK |

**Table S3**: Sequence of murine Fc-protein, related to Fc-protein production.
