# Supplementary figures and images for: Identification of FasL as a crucial host factor driving COVID-19 pathology and lethality
Source: Cell Death Differ. 2024 Mar 21;31(5):544–57. doi: 10.1038/s41418-024-01278-6 (PMC11093991; doi:10.1038/s41418-024-01278-6)

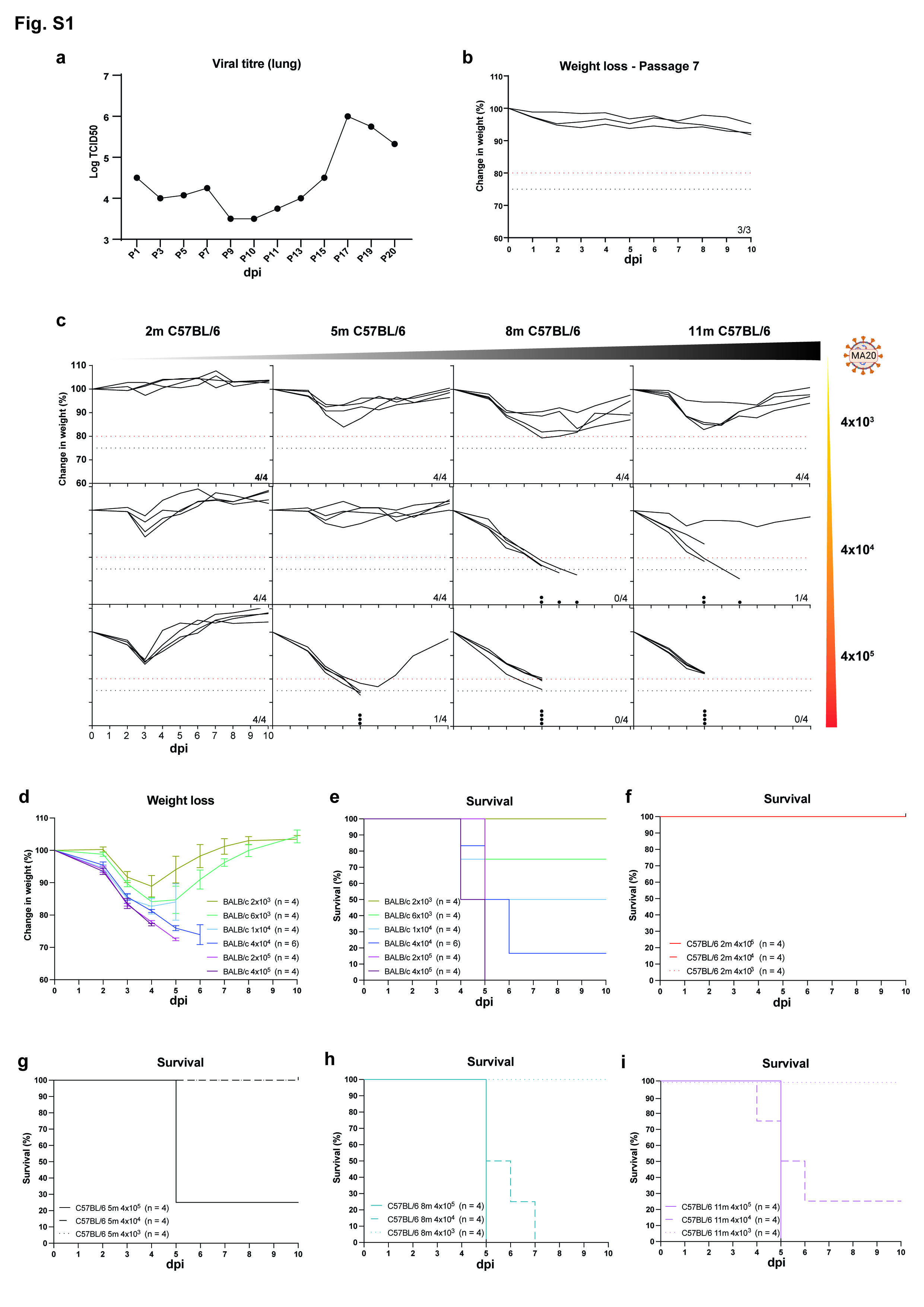

Supplement: Supplementary file 2 — Figure S1 [file 41418_2024_1278_MOESM2_ESM.jpg]

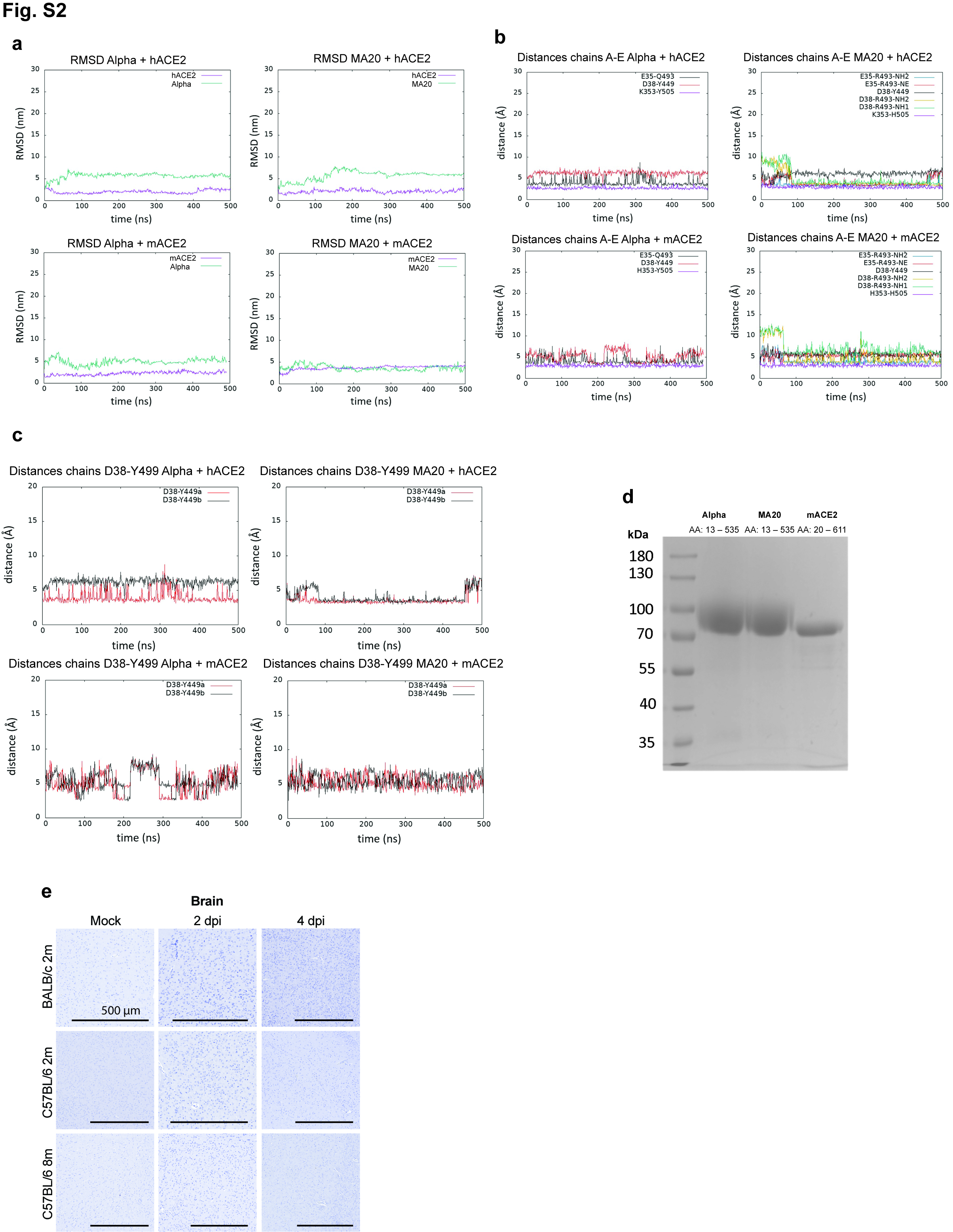

Supplement: Supplementary file 3 — Figure S2 [file 41418_2024_1278_MOESM3_ESM.jpg]

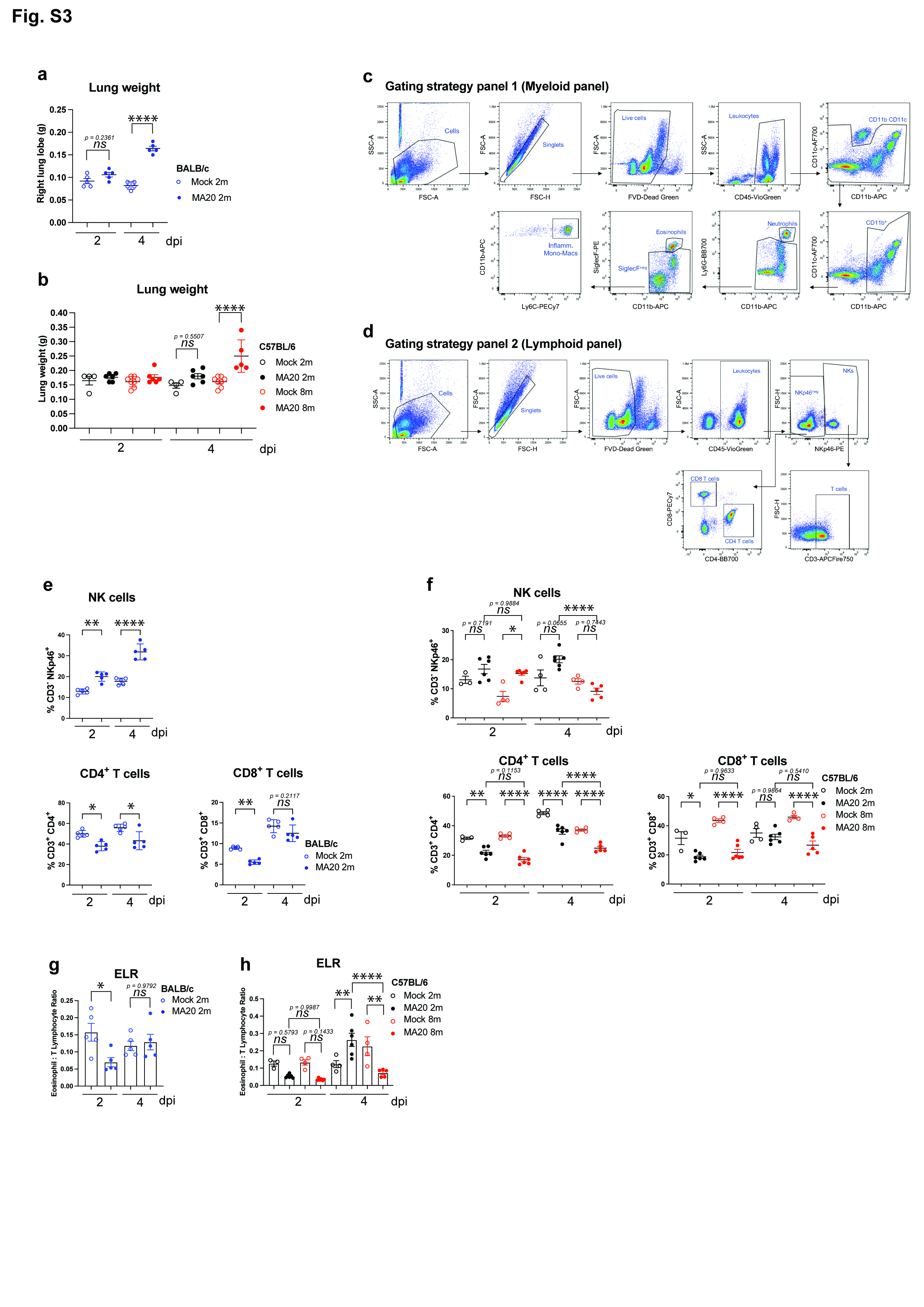

Supplement: Supplementary file 4 — Figure S3 [file 41418_2024_1278_MOESM4_ESM.jpg]

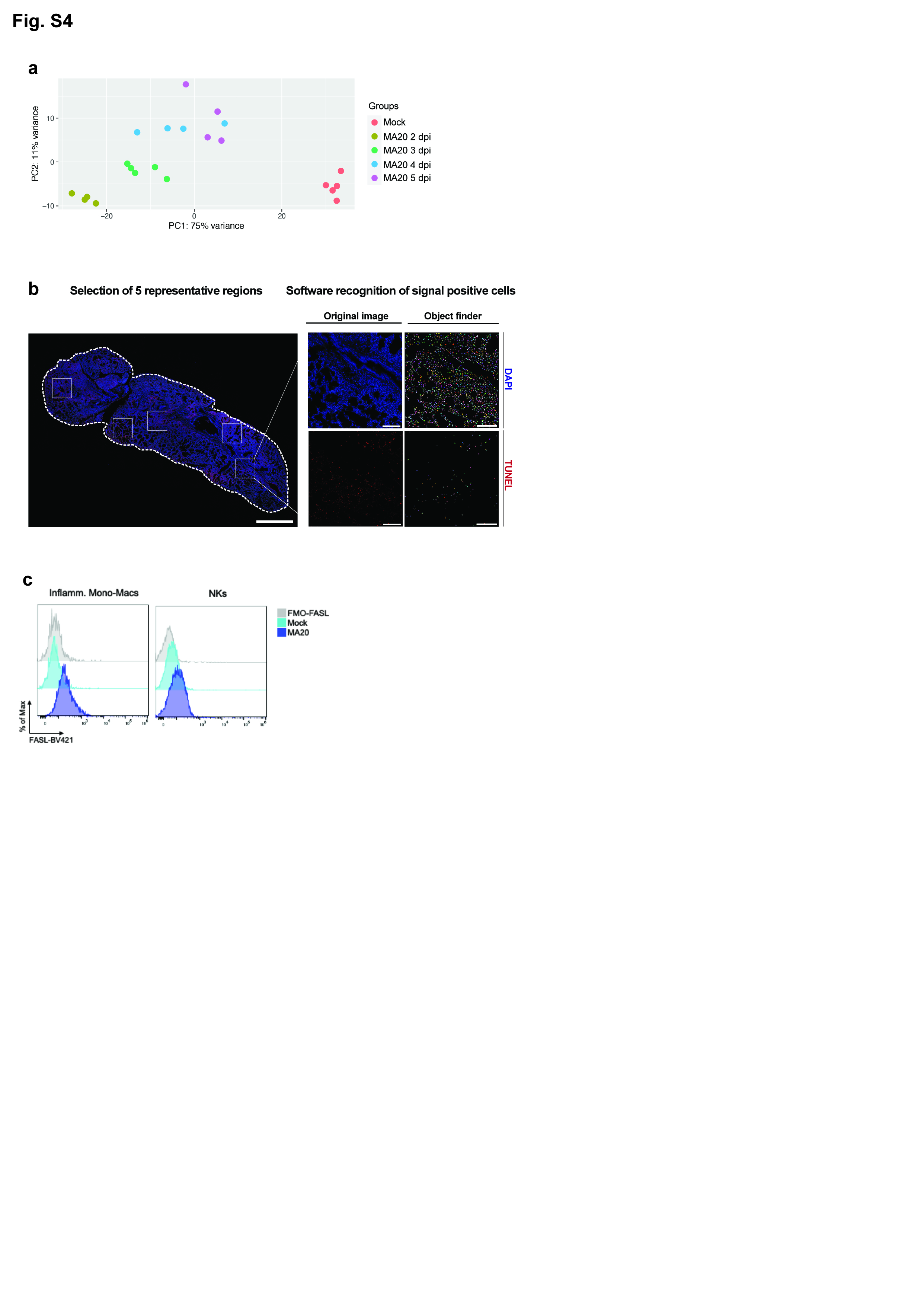

Supplement: Supplementary file 5 — Figure S4 [file 41418_2024_1278_MOESM5_ESM.jpg]

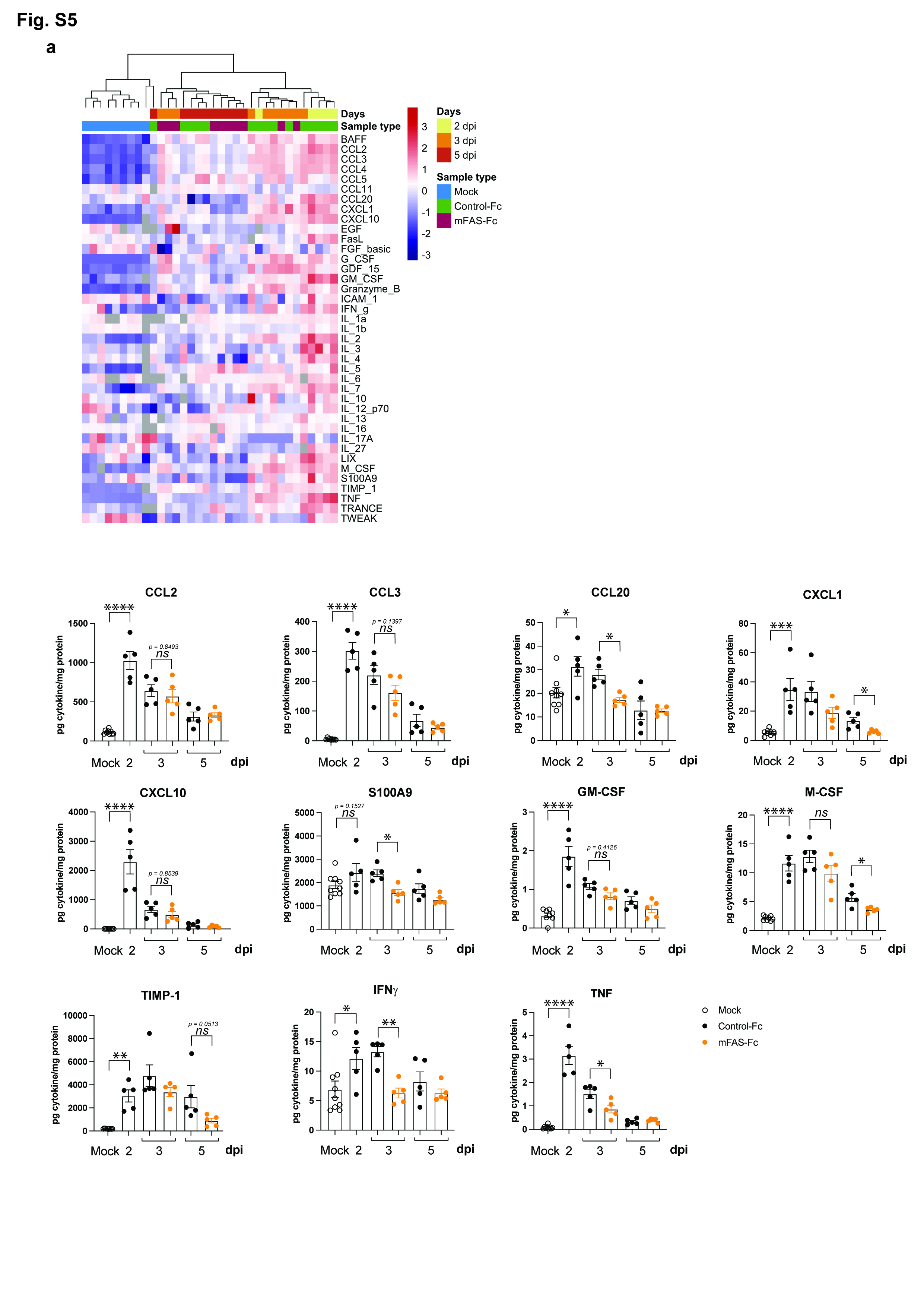

Supplement: Supplementary file 6 — Figure S5 [file 41418_2024_1278_MOESM6_ESM.jpg]
